# Supplementary material for: BICC1 interacts with PKD1 and PKD2 to drive cystogenesis in ADPKD
Source: eLife. 2026 Feb 12;14:RP106342. doi: 10.7554/eLife.106342 (PMC12900513; doi:10.7554/eLife.106342)
Supplement: Figure 1—source data 1. [file elife-106342-fig1-data1.zip › Figure 1 Source Data 1/Figure 1 Source Data 1H.pdf]

Figure 1–Source Data 1H

Figure 1 H

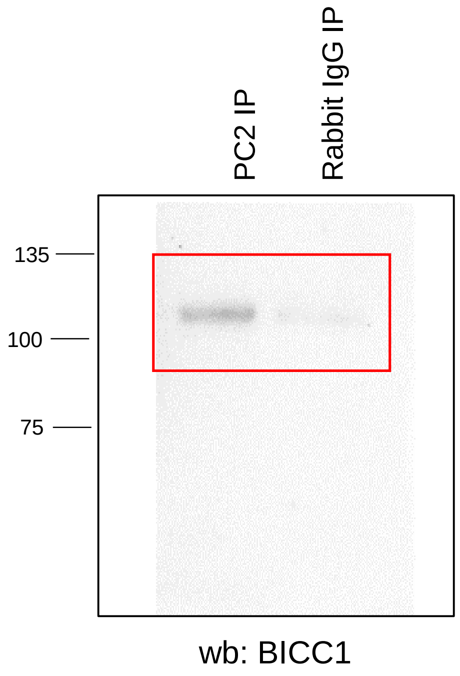

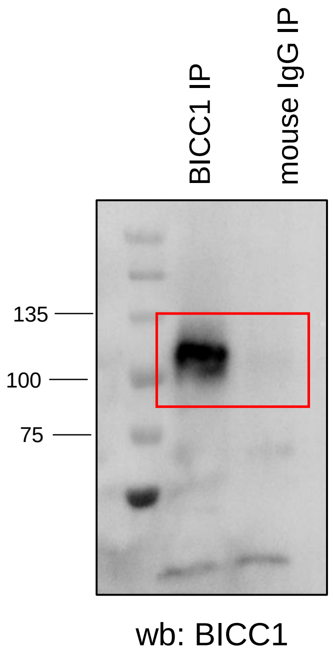

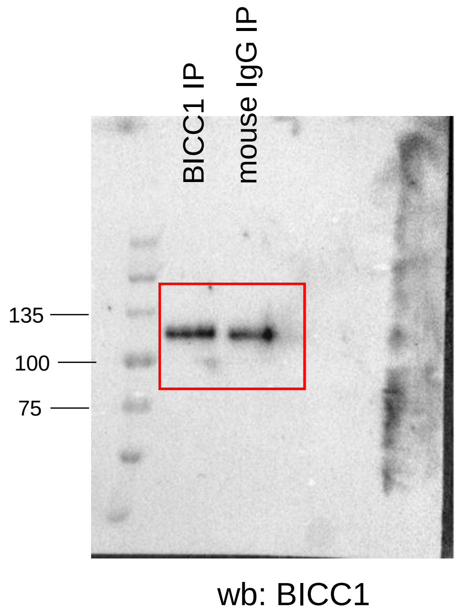

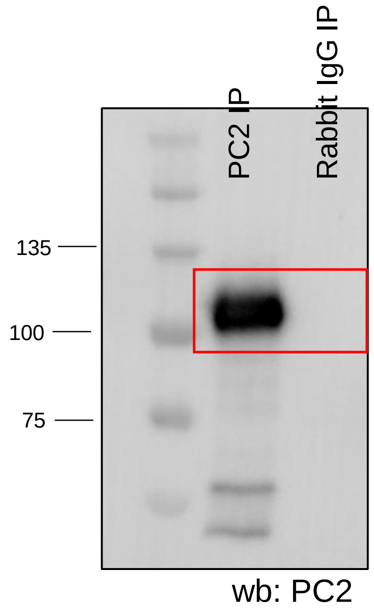

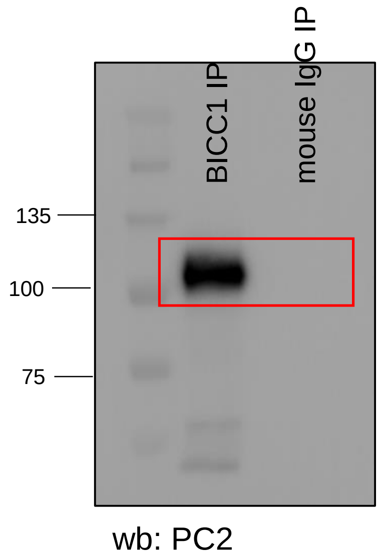

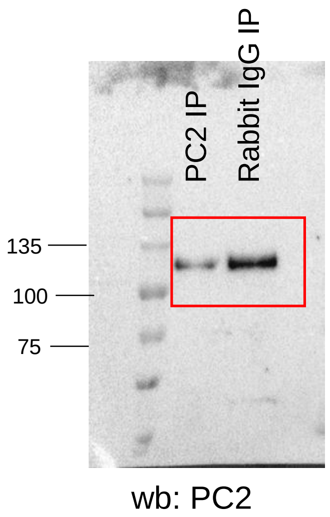

Figure 1, Source Data 1H. Original membranes corresponding to Figure 1, panel H
