# Supplementary figures and images for: BICC1 interacts with PKD1 and PKD2 to drive cystogenesis in ADPKD
Source: eLife. 2026 Feb 12;14:RP106342. doi: 10.7554/eLife.106342 (PMC12900513; doi:10.7554/eLife.106342)

Figure 1 B

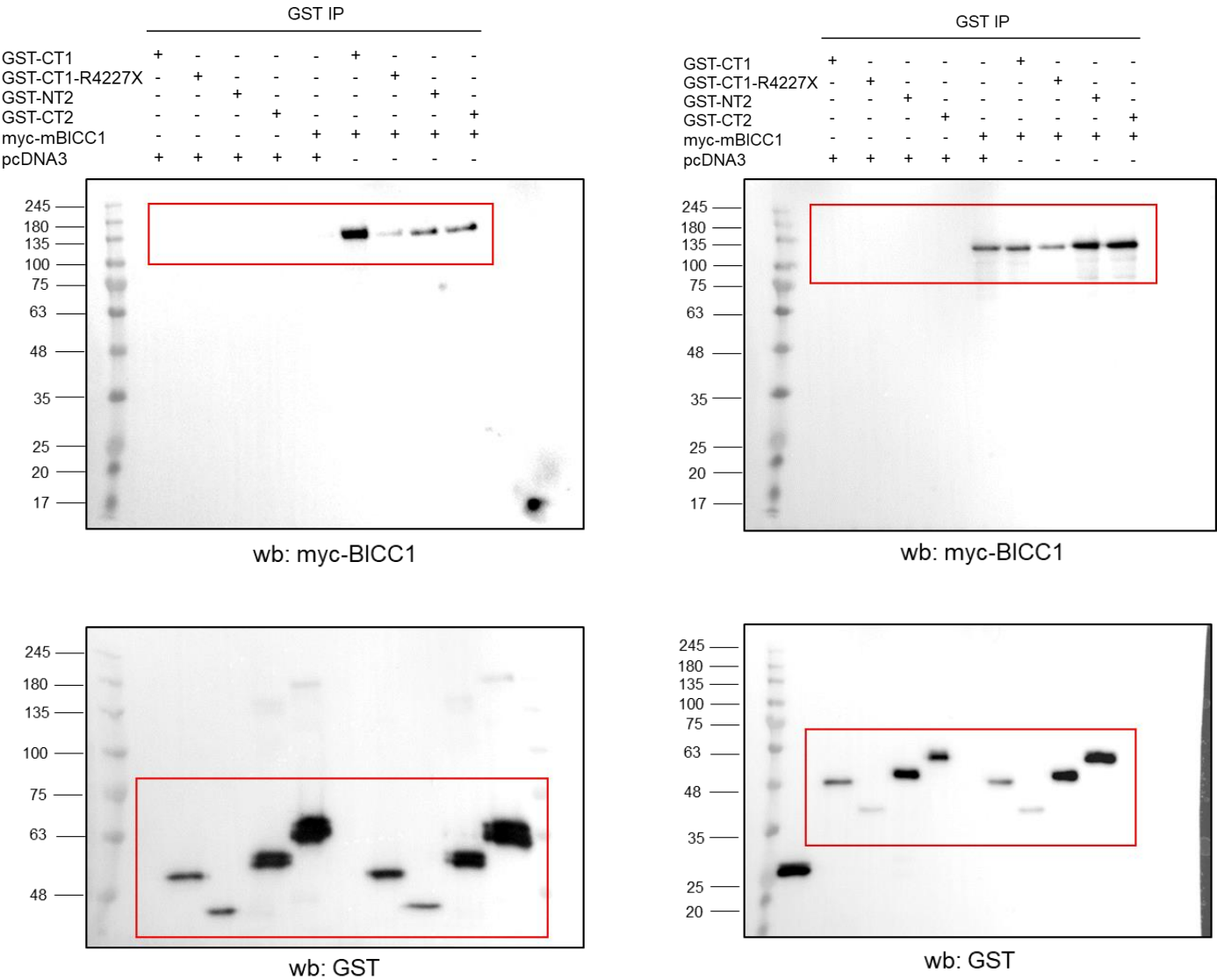

Figure 1, Source Data 1. Original membranes corresponding to Figure 1, panel B

Supplement: Figure 1—source data 1. [file elife-106342-fig1-data1.zip › Figure 1 Source Data 1/Figure 1 Source Data 1B.pdf]

Figure 1–Source Data 1C

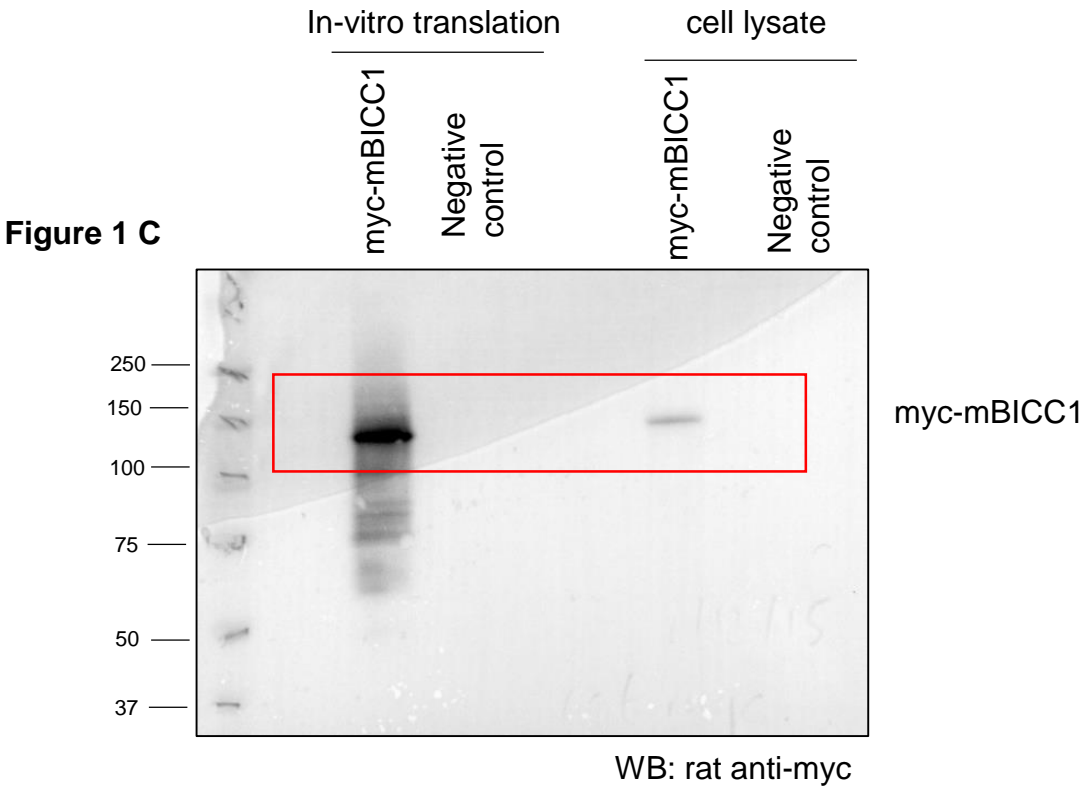

Figure 1, Source Data 1C. Original membranes corresponding to Figure 1, panel C

Supplement: Figure 1—source data 1. [file elife-106342-fig1-data1.zip › Figure 1 Source Data 1/Figure 1 Source Data 1C.pdf]

Figure 1 G

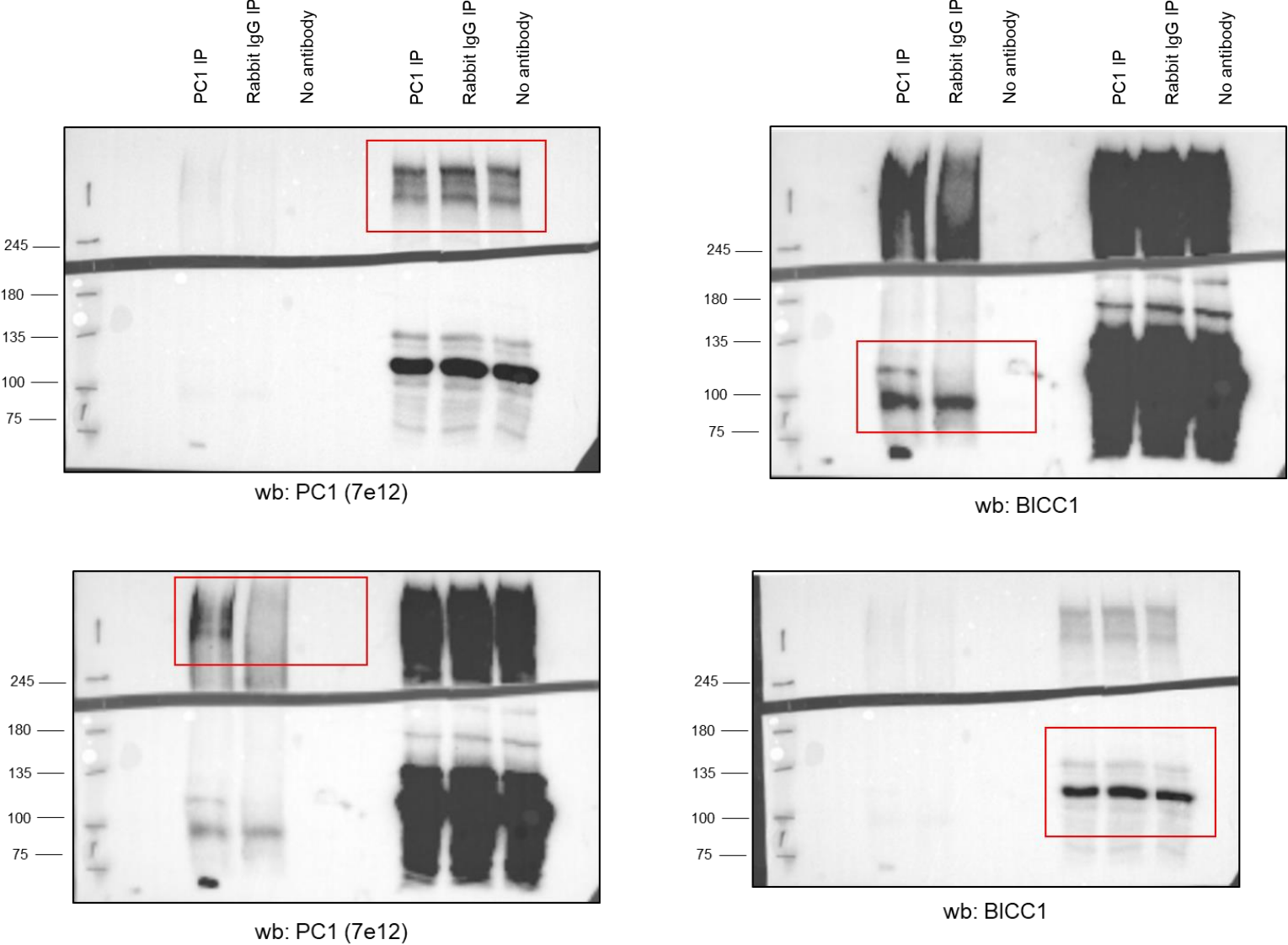

Figure 1, Source Data 1G. Original membranes corresponding to Figure 1, panel G

Supplement: Figure 1—source data 1. [file elife-106342-fig1-data1.zip › Figure 1 Source Data 1/Figure 1 Source Data 1G.pdf]

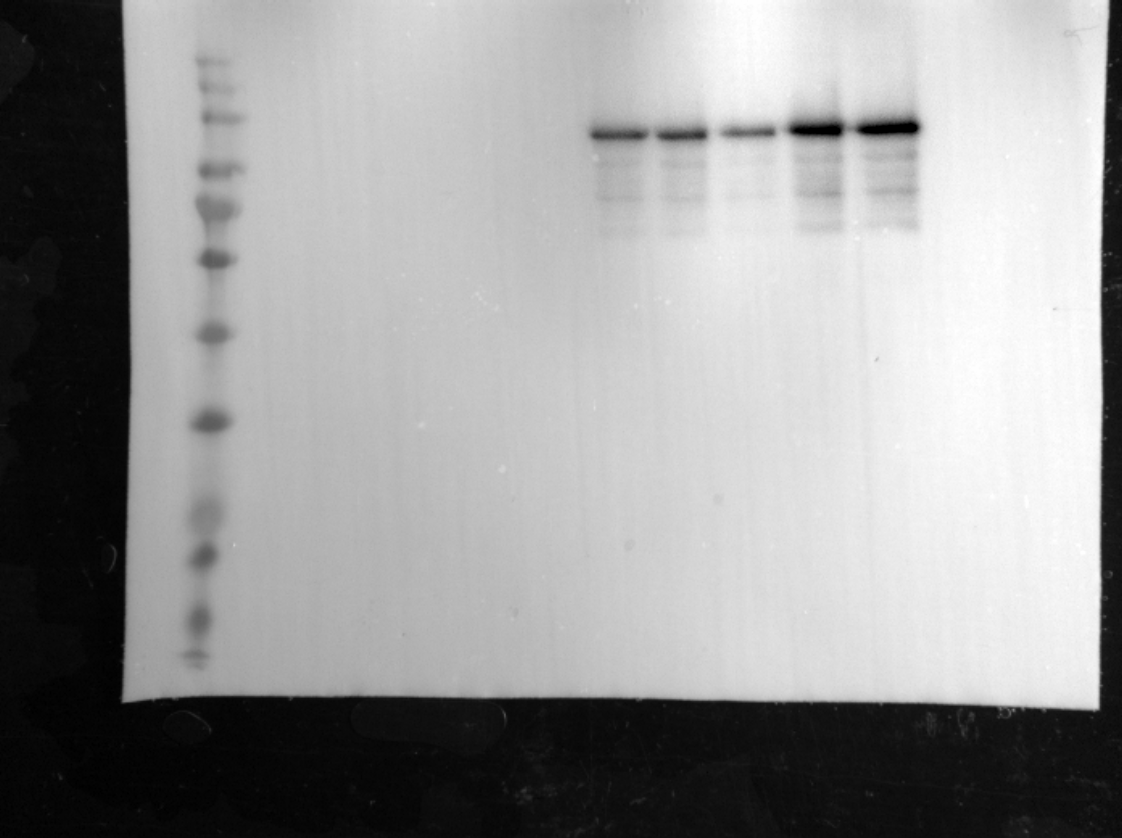

Supplement: Figure 1—source data 2. [file elife-106342-fig1-data2.zip › Figure 1 Source Data 2/Figure 1B Source Data 2/Source data Figure 1B_3.tif]

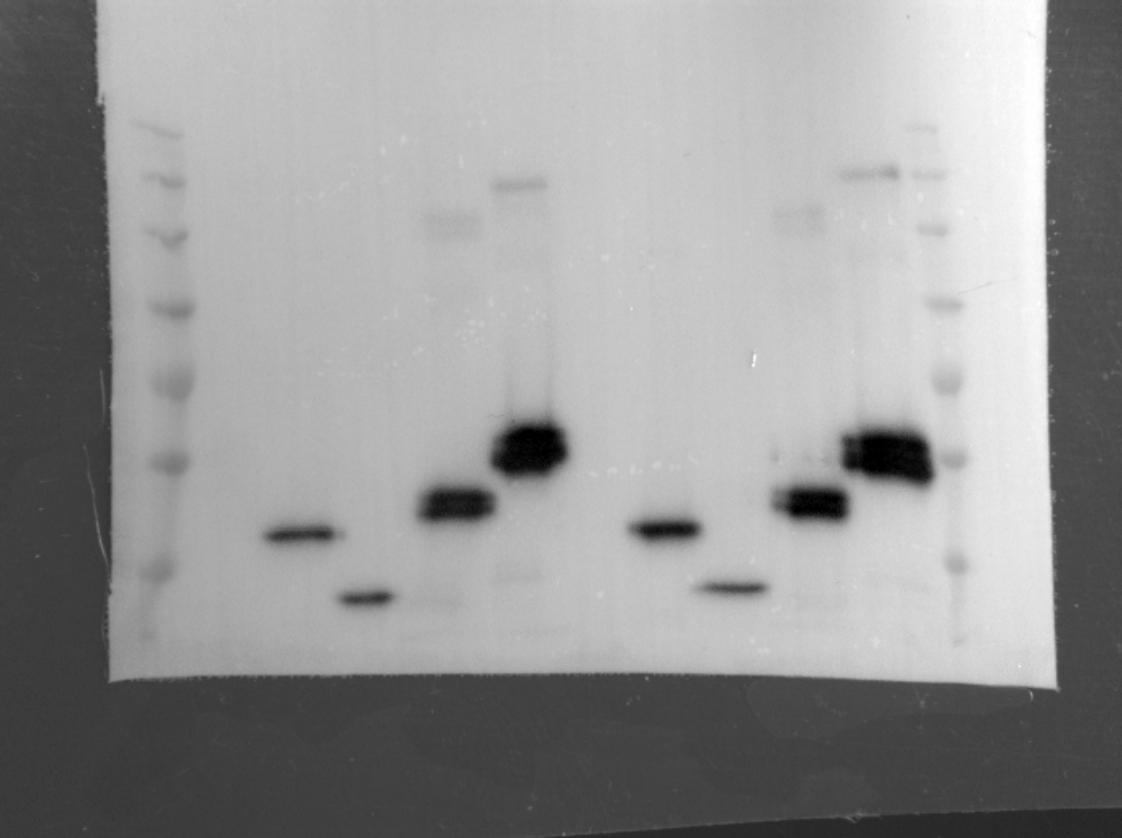

Supplement: Figure 1—source data 2. [file elife-106342-fig1-data2.zip › Figure 1 Source Data 2/Figure 1B Source Data 2/Source data Figure 1B_2.tif]

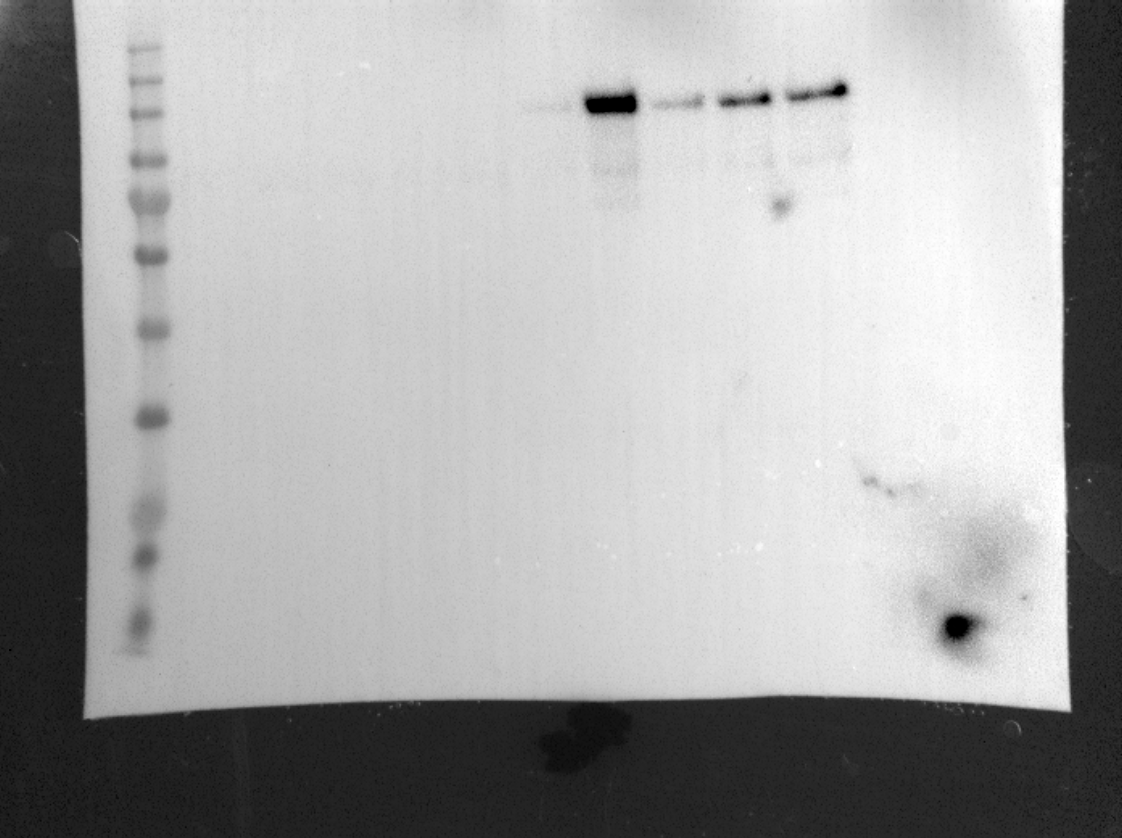

Supplement: Figure 1—source data 2. [file elife-106342-fig1-data2.zip › Figure 1 Source Data 2/Figure 1B Source Data 2/Source data Figure 1B_1.tif]

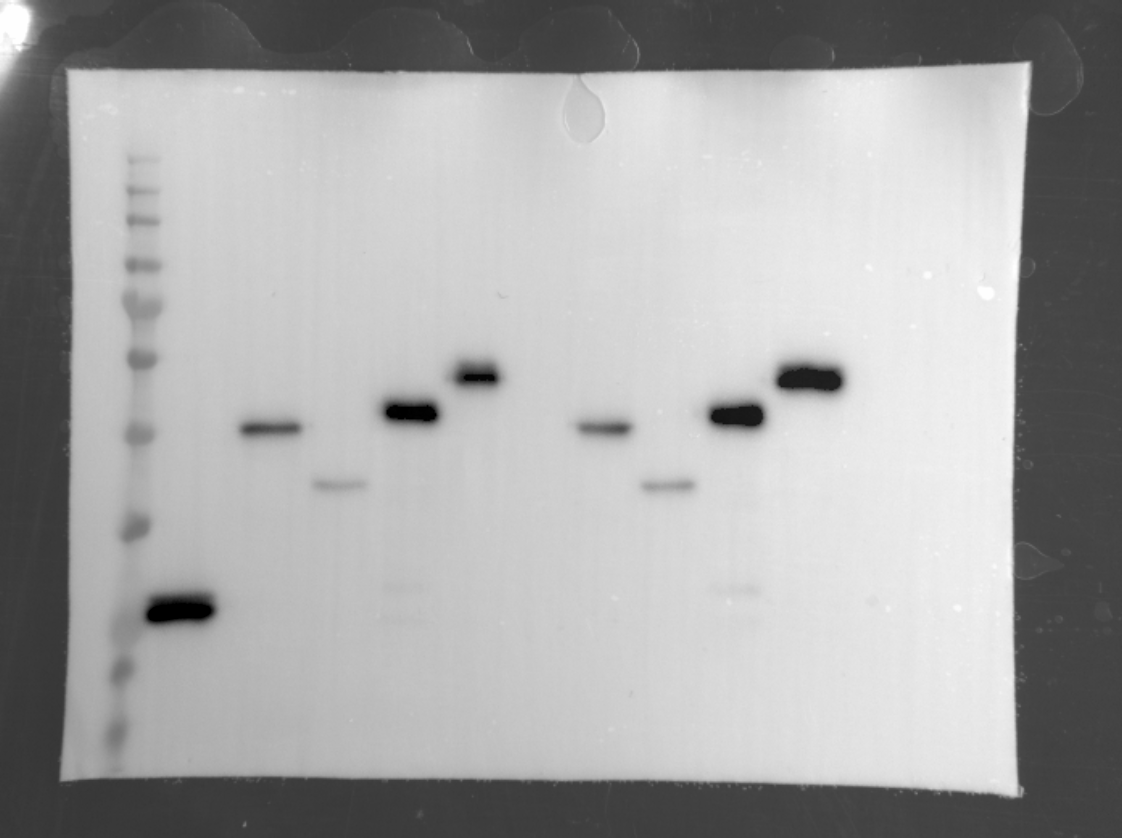

Supplement: Figure 1—source data 2. [file elife-106342-fig1-data2.zip › Figure 1 Source Data 2/Figure 1B Source Data 2/Source data Figure 1B_4.tif]

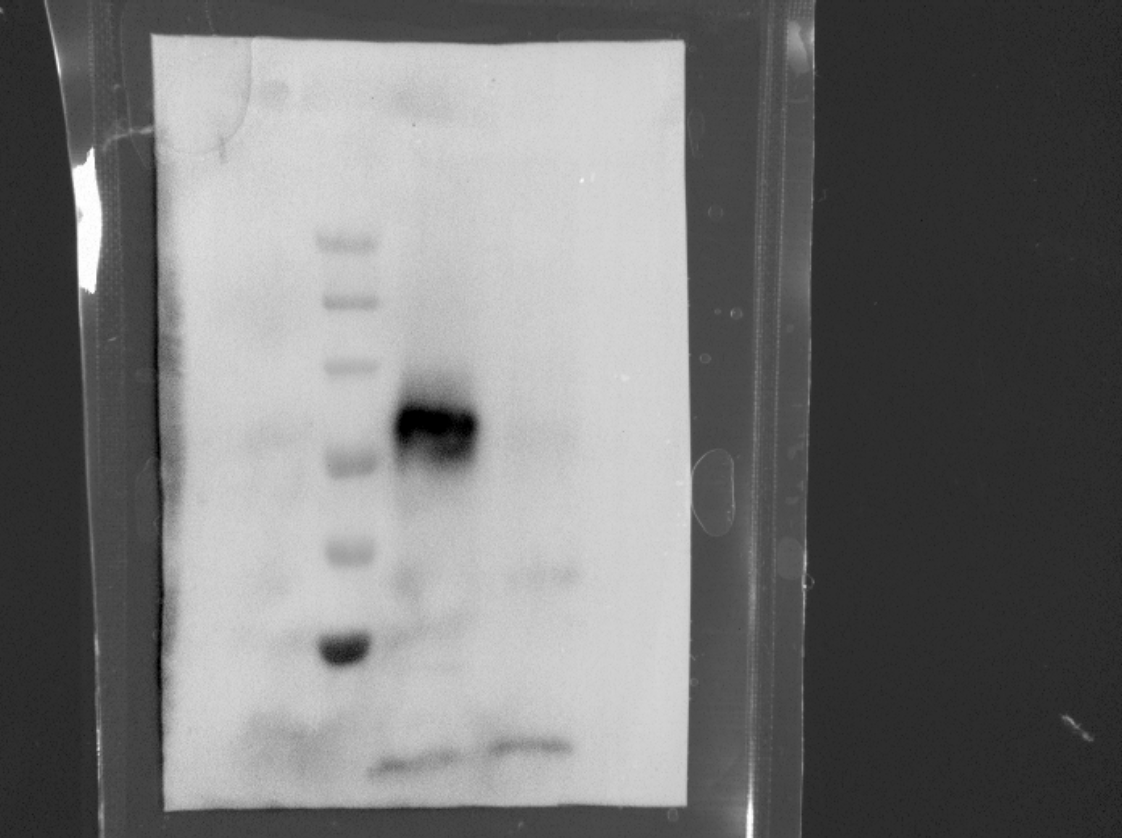

Supplement: Figure 1—source data 2. [file elife-106342-fig1-data2.zip › Figure 1 Source Data 2/Figure 1H Source Data 2/Source Figure 1H_3.tif]

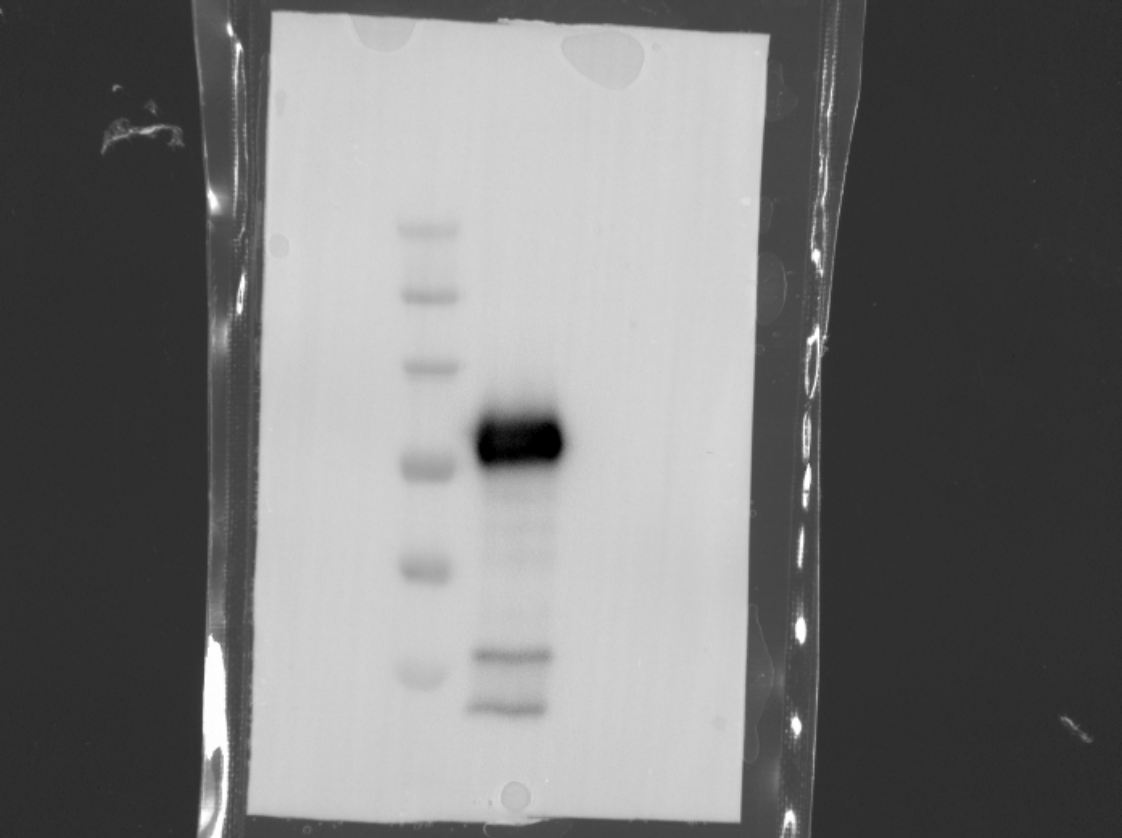

Supplement: Figure 1—source data 2. [file elife-106342-fig1-data2.zip › Figure 1 Source Data 2/Figure 1H Source Data 2/Source Figure 1H_2.tif]

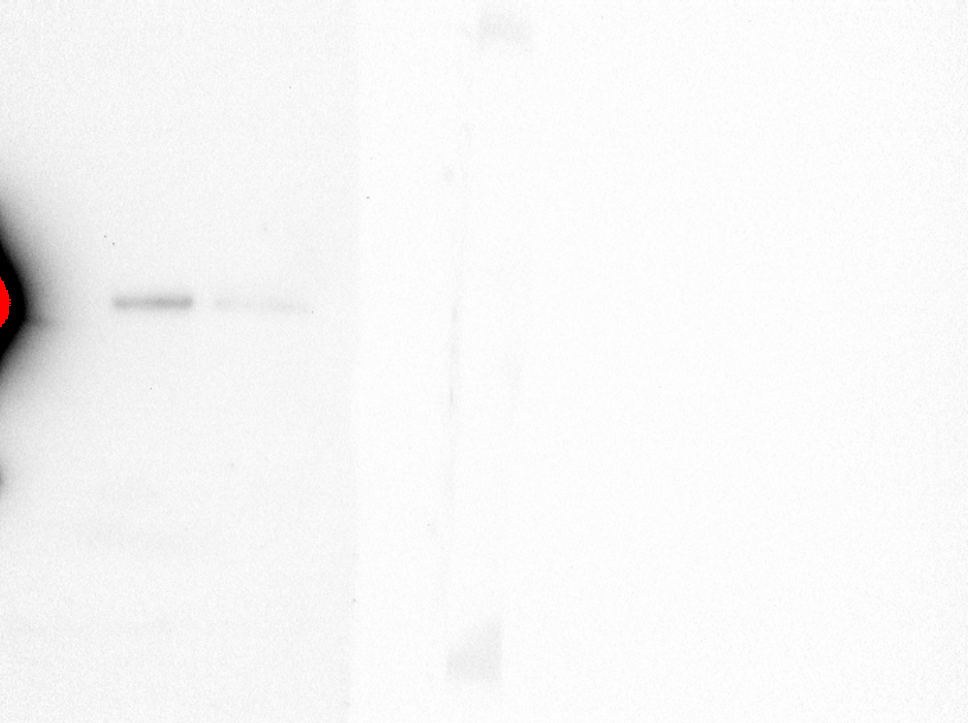

Supplement: Figure 1—source data 2. [file elife-106342-fig1-data2.zip › Figure 1 Source Data 2/Figure 1H Source Data 2/Source Figure 1H_1.tif]

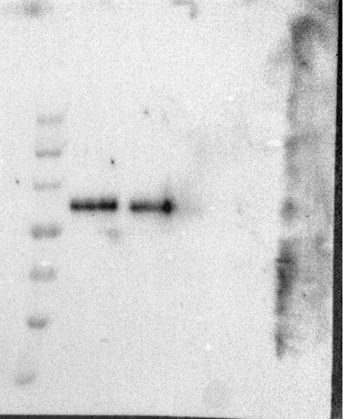

Supplement: Figure 1—source data 2. [file elife-106342-fig1-data2.zip › Figure 1 Source Data 2/Figure 1H Source Data 2/Source Figure 1H_5.tif]

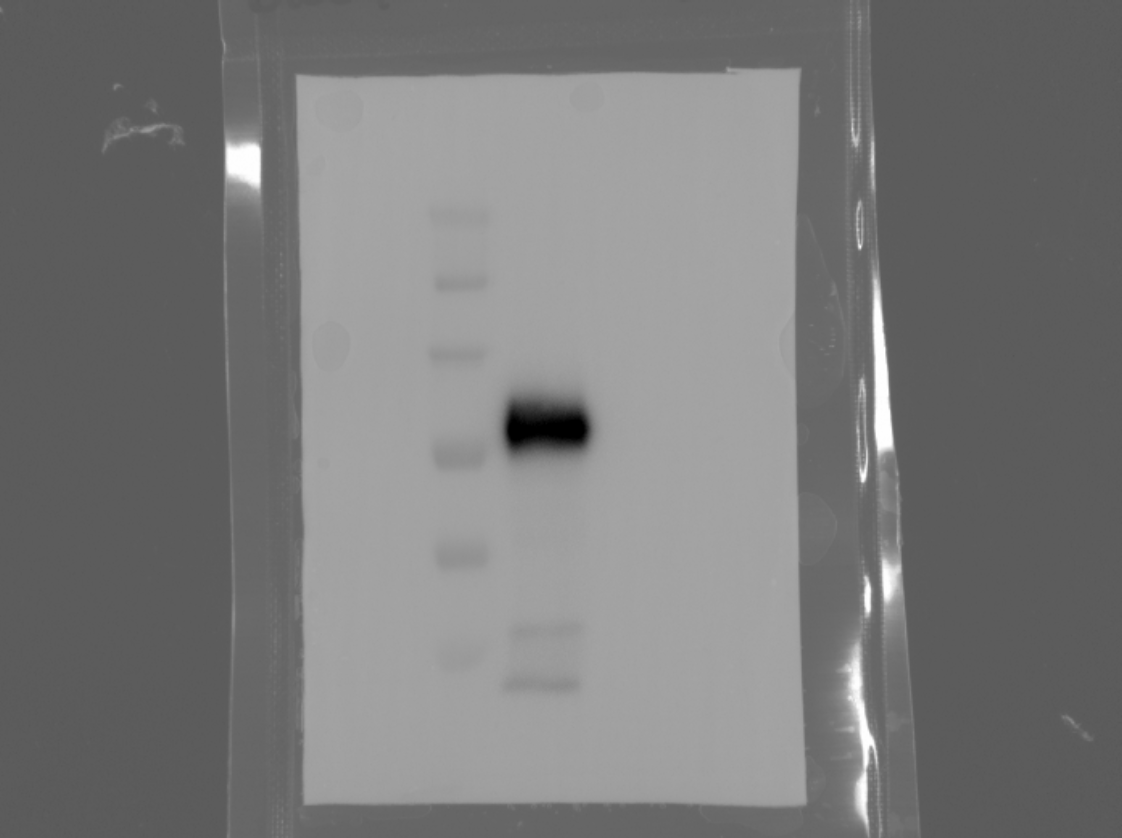

Supplement: Figure 1—source data 2. [file elife-106342-fig1-data2.zip › Figure 1 Source Data 2/Figure 1H Source Data 2/Source Figure 1H_4.tif]

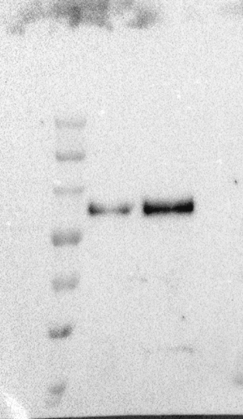

Supplement: Figure 1—source data 2. [file elife-106342-fig1-data2.zip › Figure 1 Source Data 2/Figure 1H Source Data 2/Source Figure 1H_6.tif]

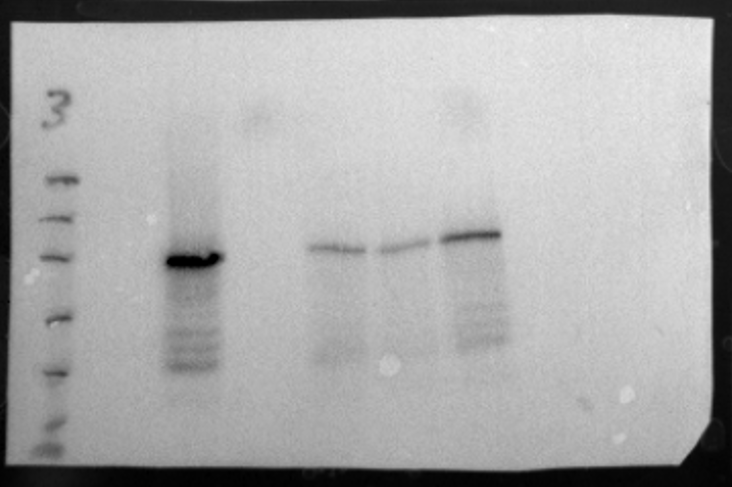

Supplement: Figure 1—source data 2. [file elife-106342-fig1-data2.zip › Figure 1 Source Data 2/Figure 1D Source Data 2/Source data Figure 1D myc_BICC1_1.tif]

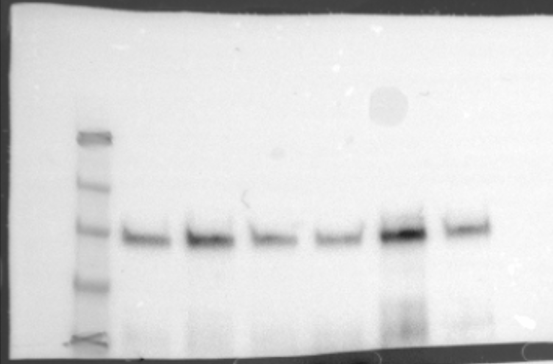

Supplement: Figure 1—source data 2. [file elife-106342-fig1-data2.zip › Figure 1 Source Data 2/Figure 1D Source Data 2/Source data Figure 1D myc_BICC1_2.tif]

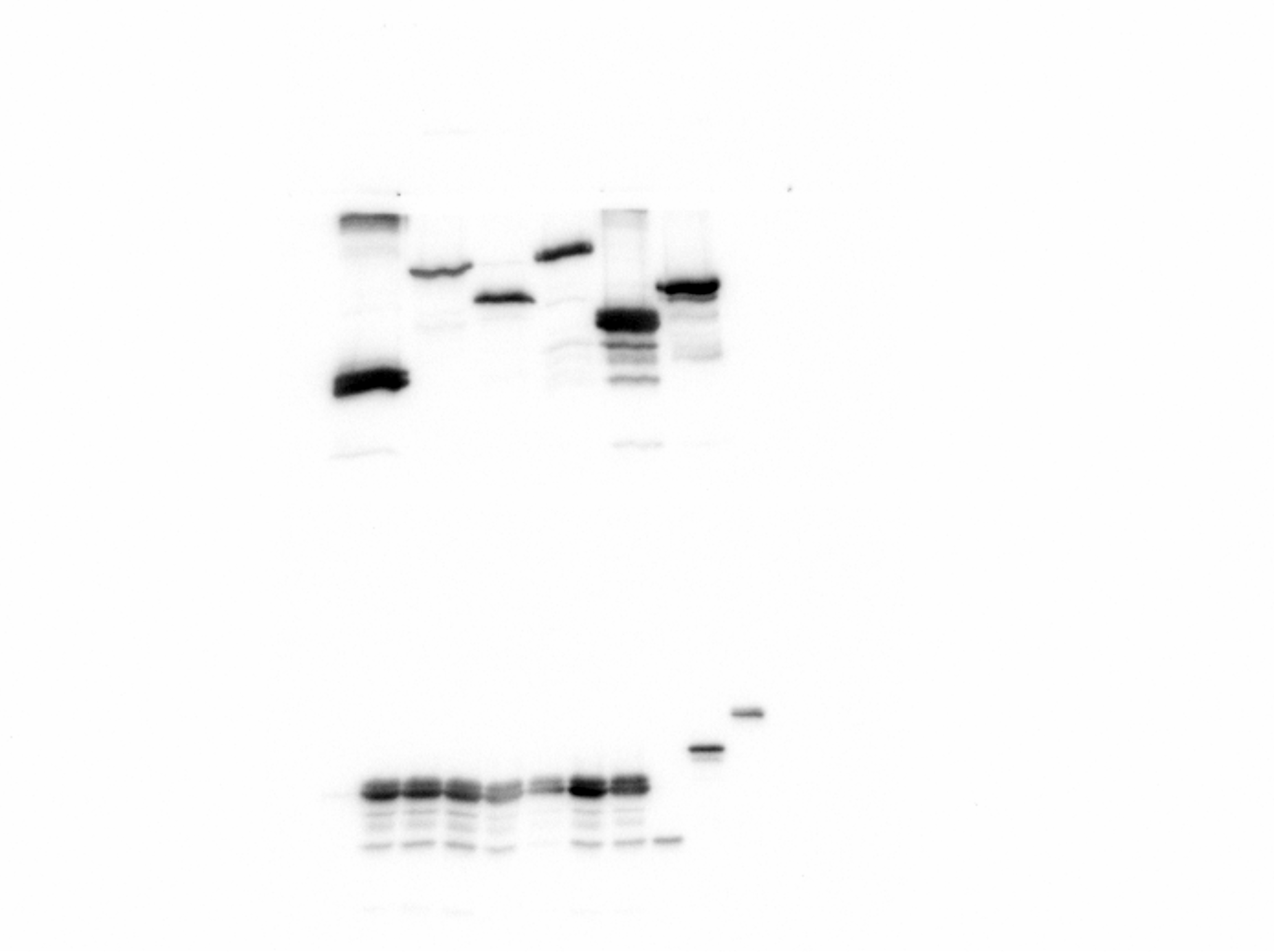

Supplement: Figure 1—source data 2. [file elife-106342-fig1-data2.zip › Figure 1 Source Data 2/Figure 1D Source Data 2/Source data Figure 1D GST.tif]

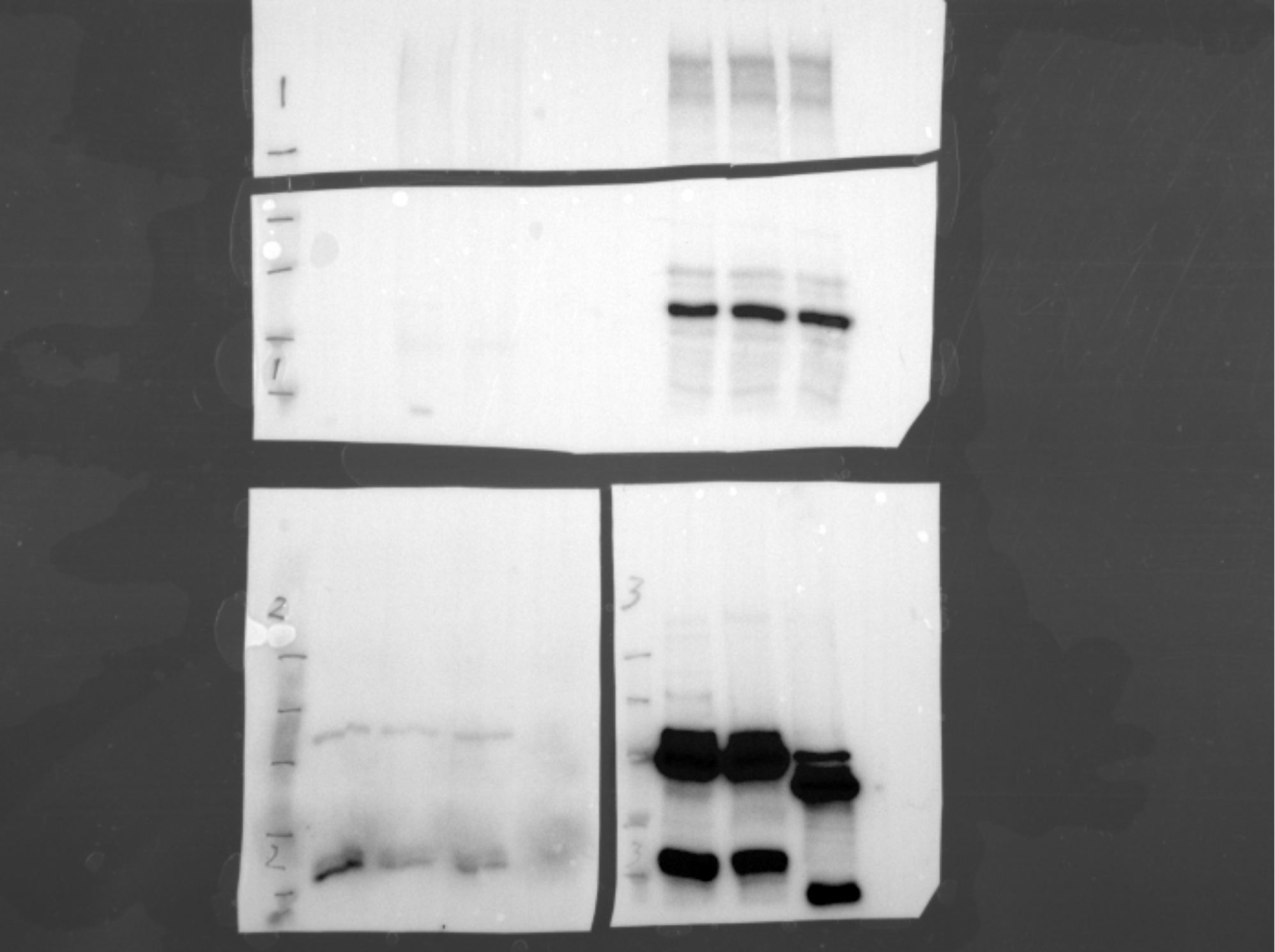

Supplement: Figure 1—source data 2. [file elife-106342-fig1-data2.zip › Figure 1 Source Data 2/Figure 1G Source Data 2/Source data Figure 1G_4.tif]

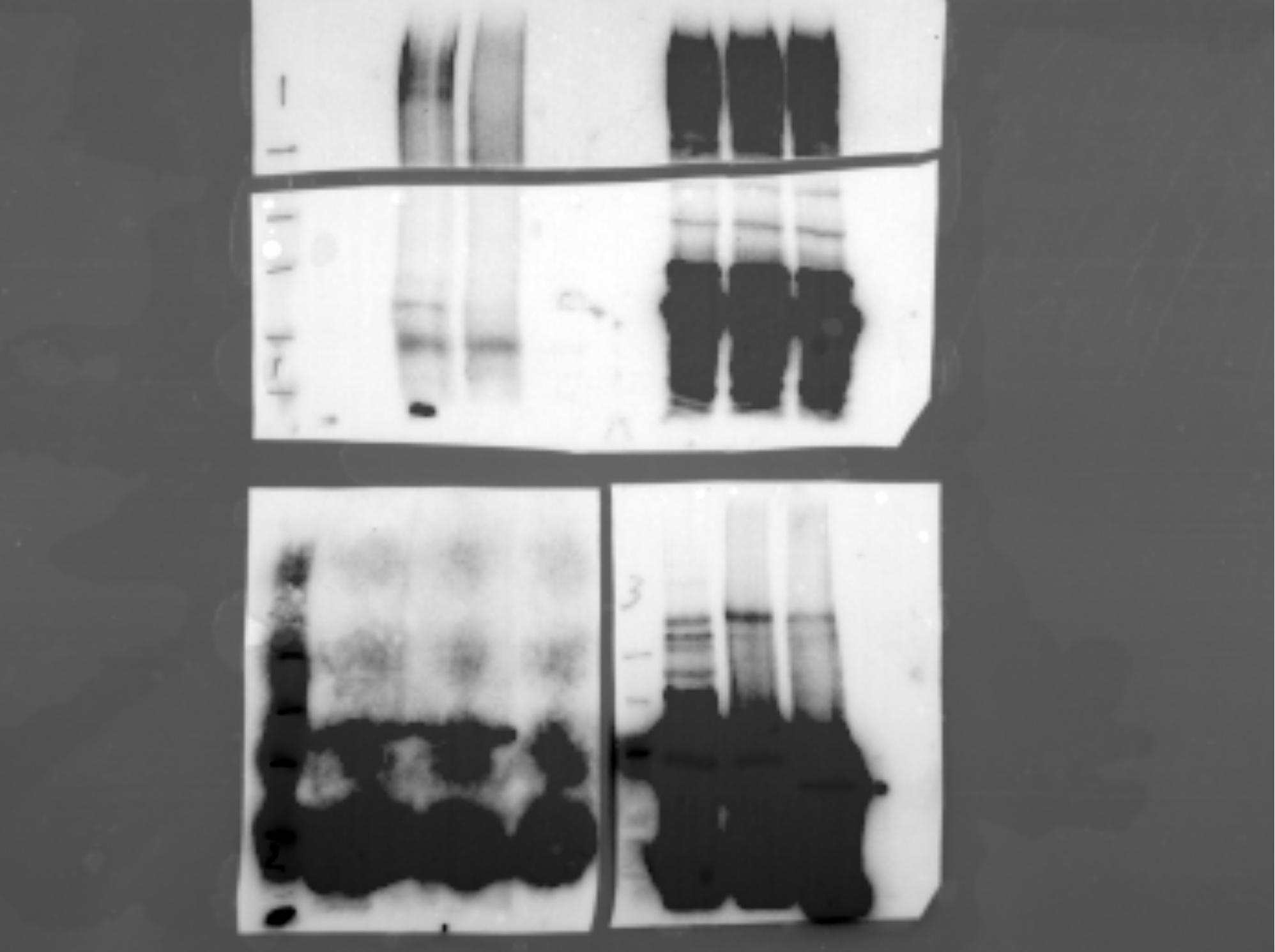

Supplement: Figure 1—source data 2. [file elife-106342-fig1-data2.zip › Figure 1 Source Data 2/Figure 1G Source Data 2/Source data Figure 1G_2.tif]

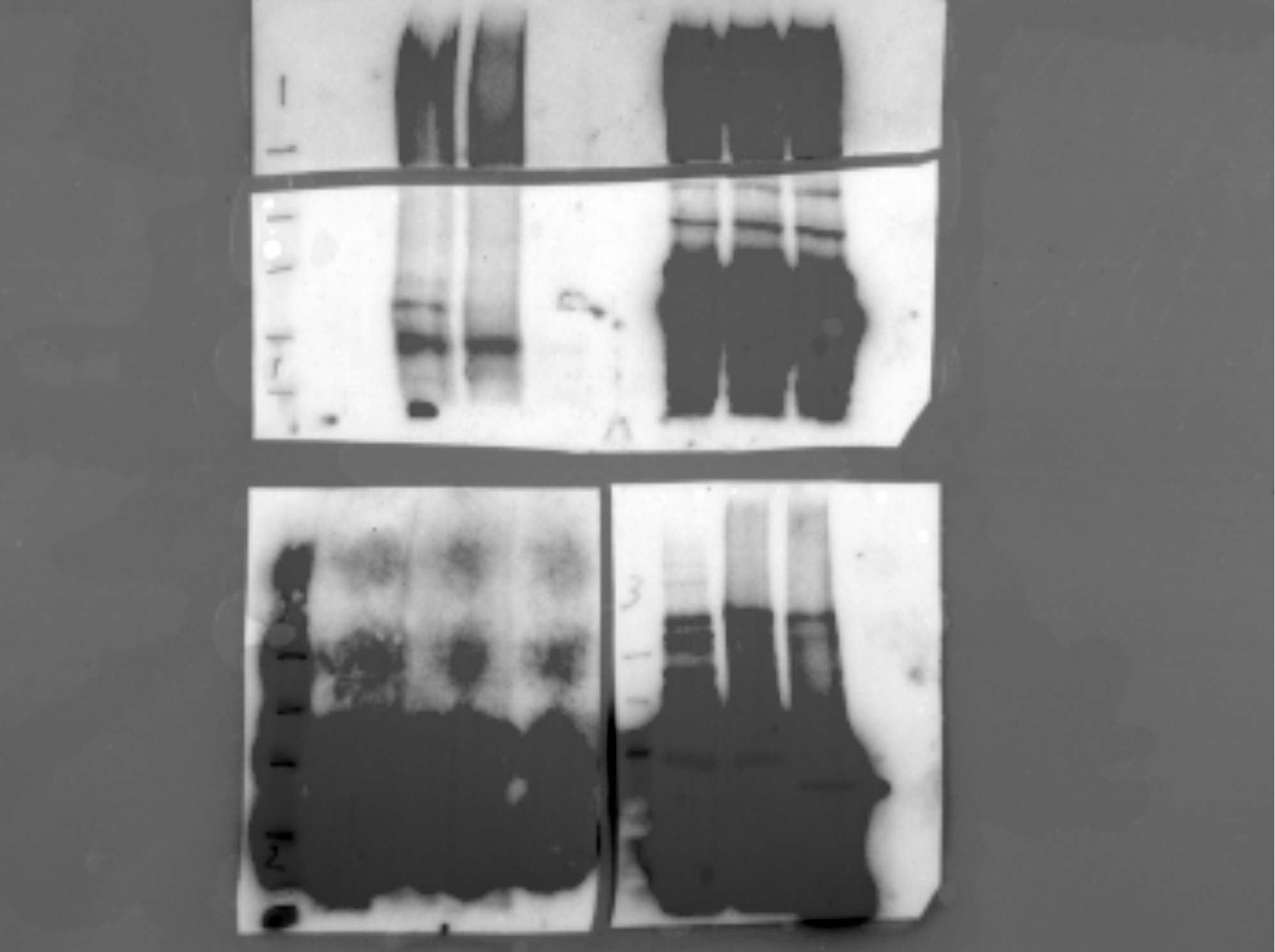

Supplement: Figure 1—source data 2. [file elife-106342-fig1-data2.zip › Figure 1 Source Data 2/Figure 1G Source Data 2/Source data Figure 1G_3.tif]

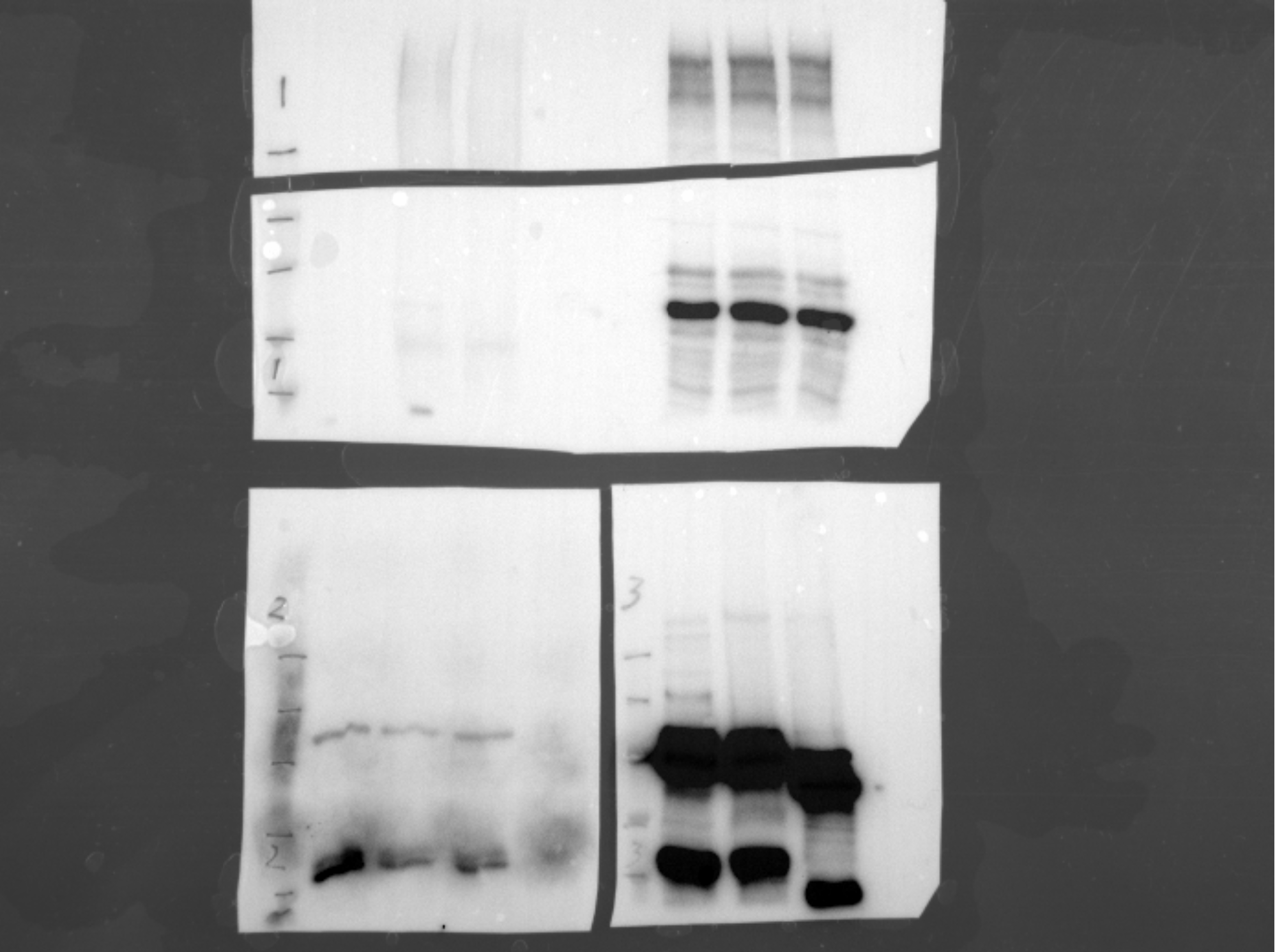

Supplement: Figure 1—source data 2. [file elife-106342-fig1-data2.zip › Figure 1 Source Data 2/Figure 1G Source Data 2/Source data Figure 1G_1.tif]

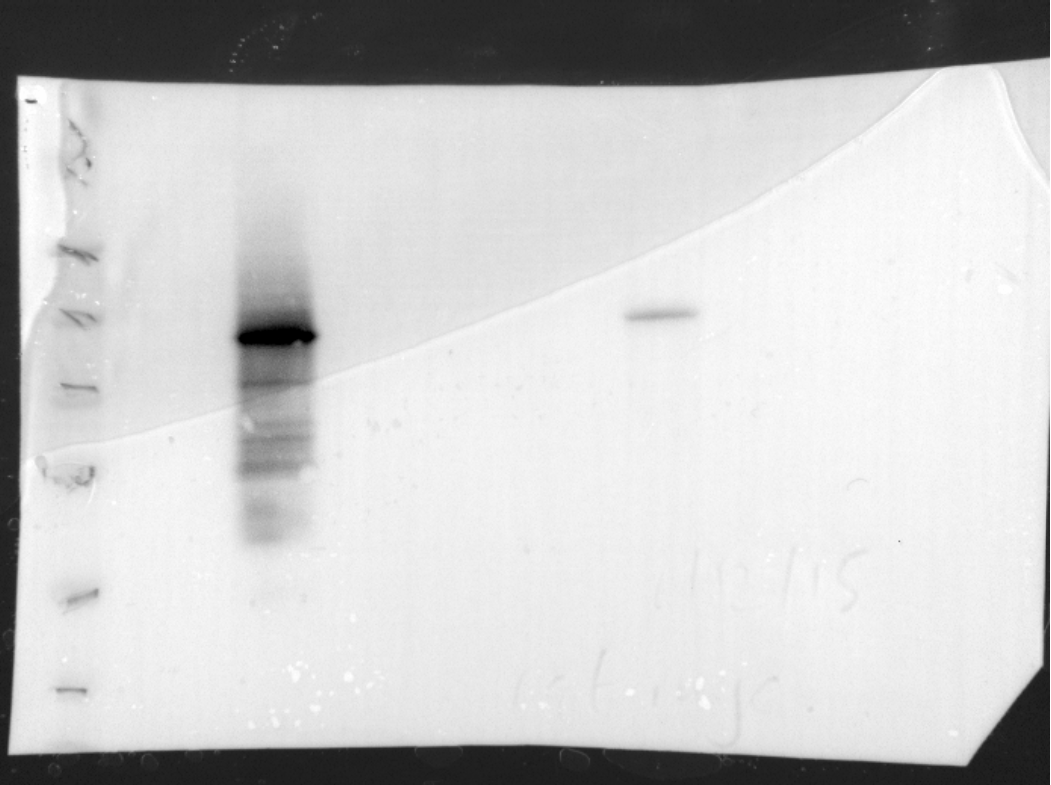

Supplement: Figure 1—source data 2. [file elife-106342-fig1-data2.zip › Figure 1 Source Data 2/Figure 1C Source Data 2/Source data Figure 1C myc_BICC1.tif]

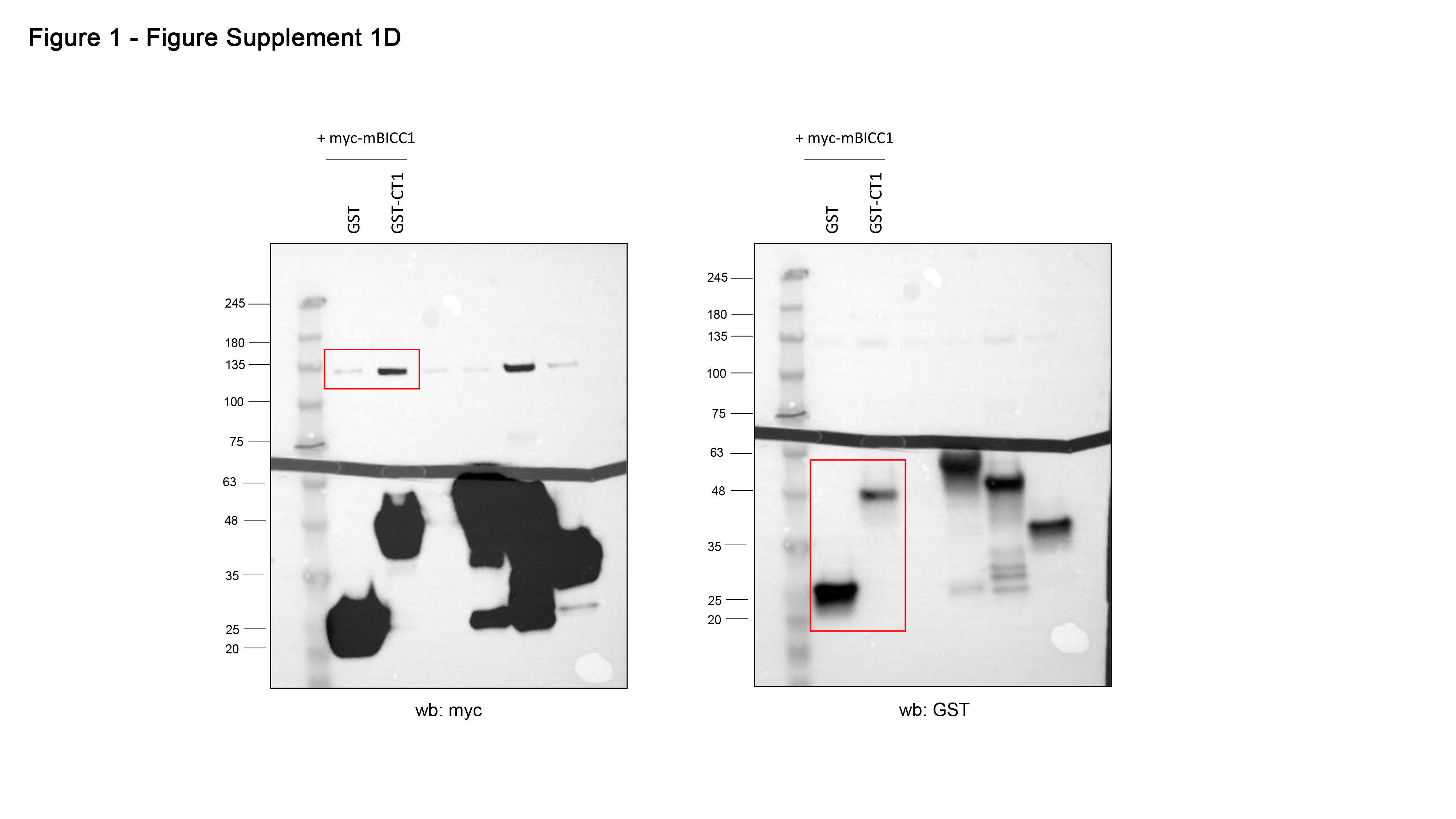

Supplement: Figure 1—figure supplement 1—source data 1. [file elife-106342-fig1-figsupp1-data1.zip › Figure 1 - Figure Supplement 1 Source data 1/Figure 1 - Figure Supplement 1D.tiff]

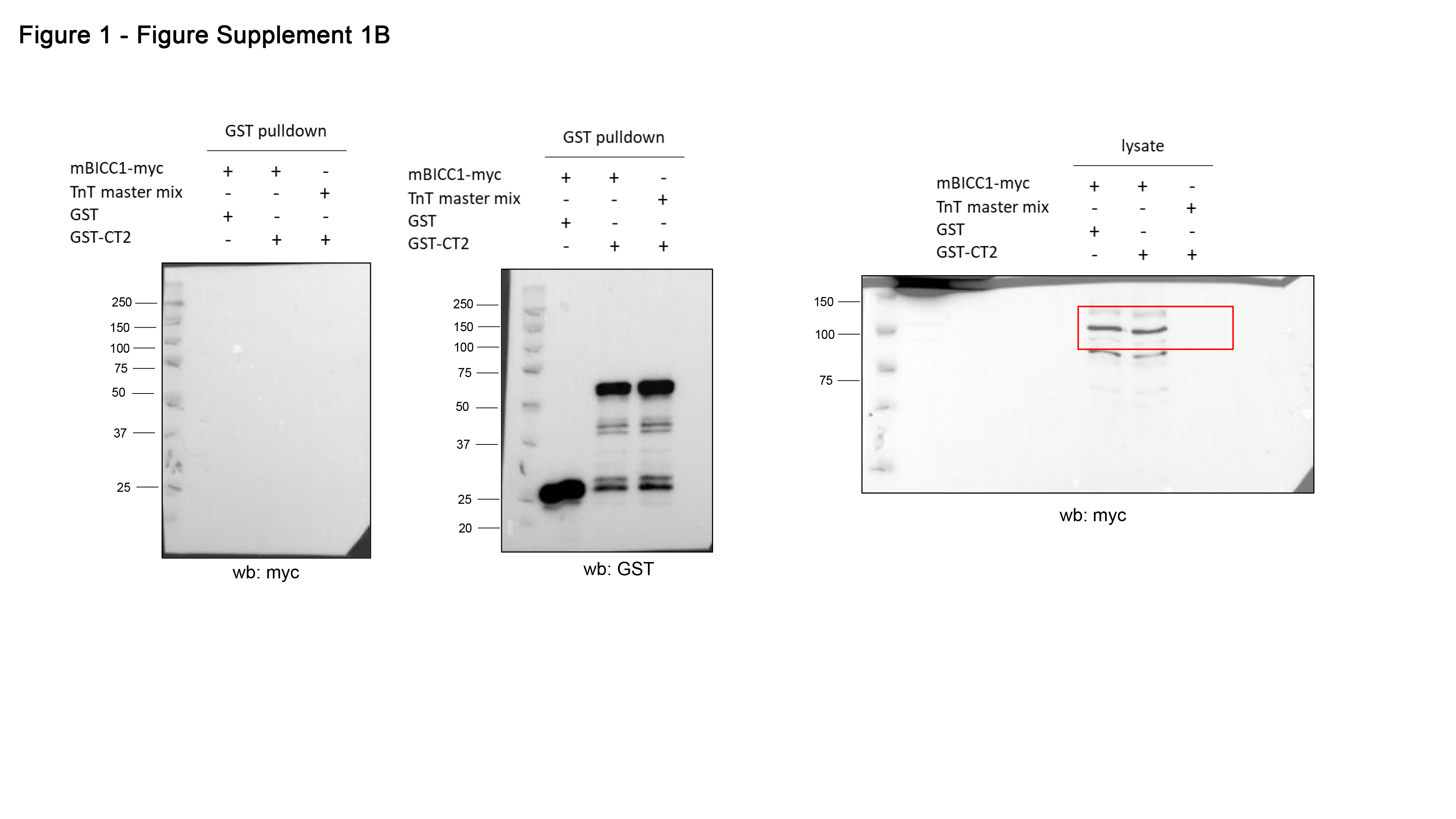

Supplement: Figure 1—figure supplement 1—source data 1. [file elife-106342-fig1-figsupp1-data1.zip › Figure 1 - Figure Supplement 1 Source data 1/Figure 1 - Figure Supplement 1B.tiff]

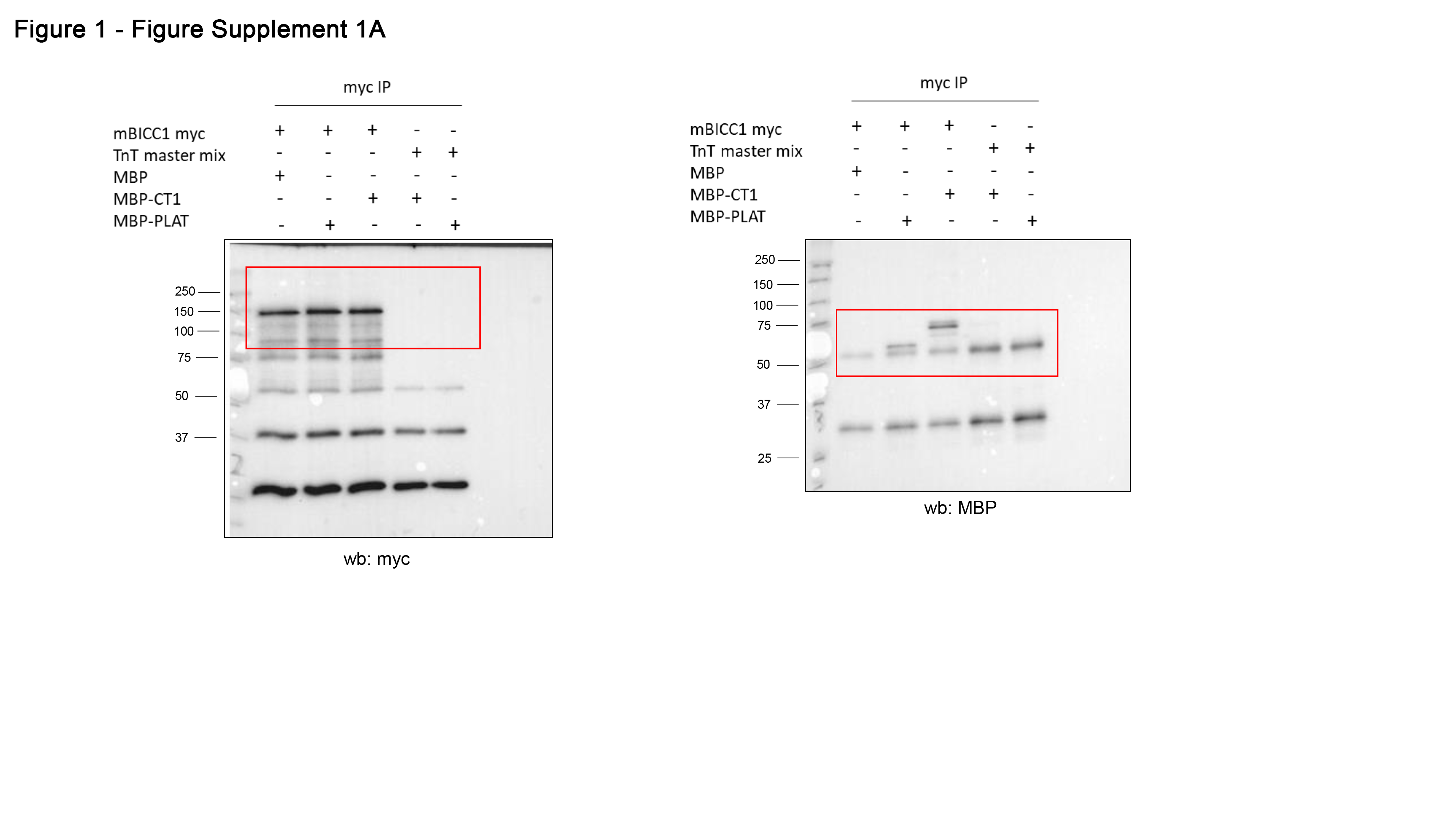

Supplement: Figure 1—figure supplement 1—source data 1. [file elife-106342-fig1-figsupp1-data1.zip › Figure 1 - Figure Supplement 1 Source data 1/Figure 1 - Figure Supplement 1A.tiff]

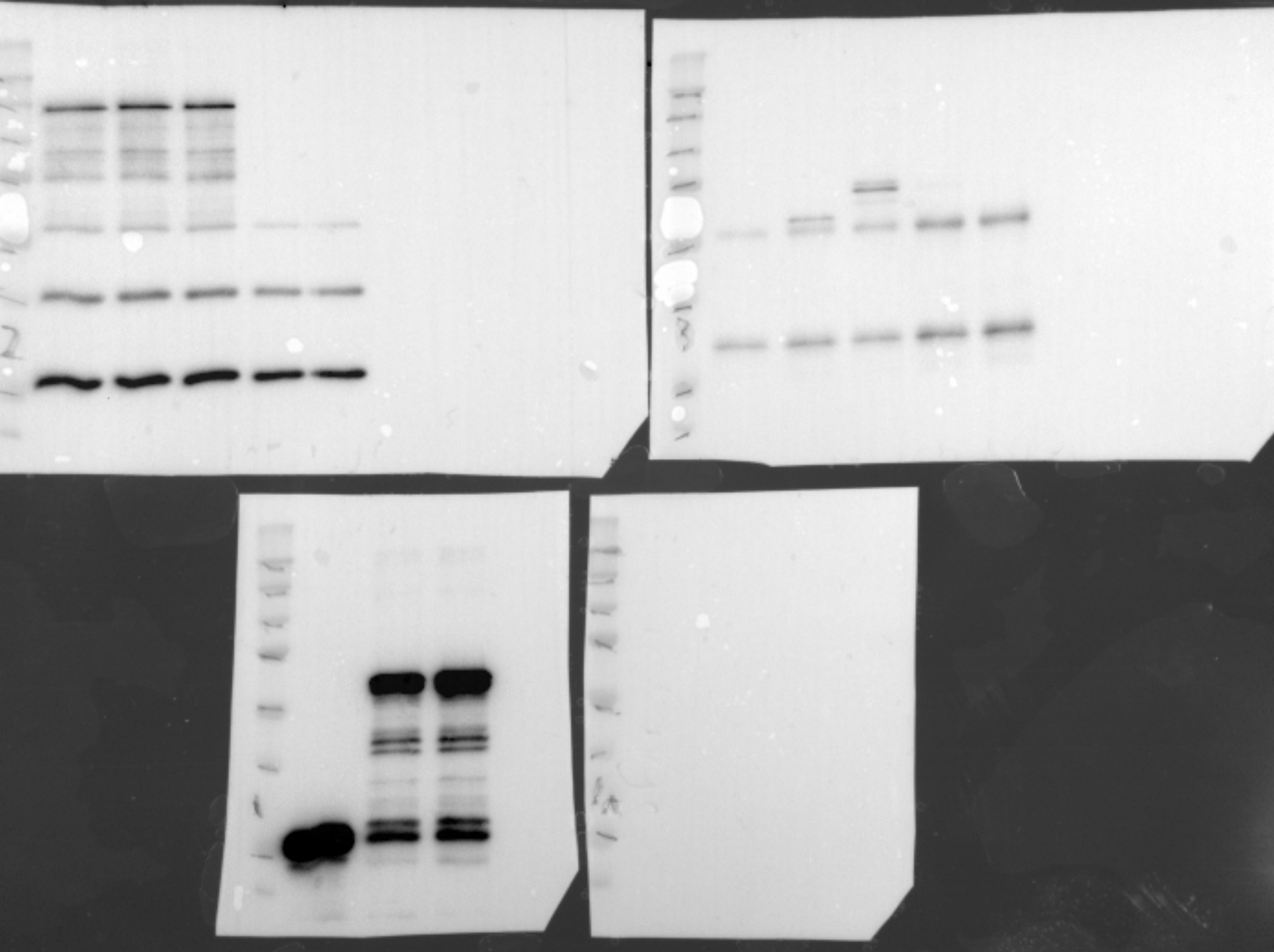

Supplement: Figure 1—figure supplement 1—source data 2. [file elife-106342-fig1-figsupp1-data2.zip › Figure 1 - Figure Supplement 1 Source data 2/Figure 1 - Figure Supplement 1B - Source Data 2/Figure 1 - Figure Supplement 1B - GST_myc.tif]

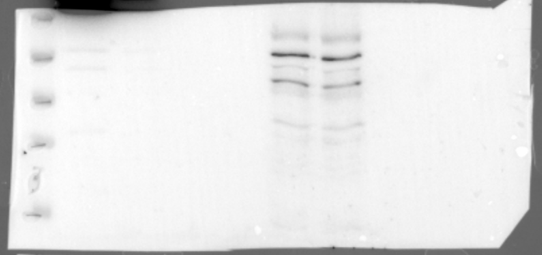

Supplement: Figure 1—figure supplement 1—source data 2. [file elife-106342-fig1-figsupp1-data2.zip › Figure 1 - Figure Supplement 1 Source data 2/Figure 1 - Figure Supplement 1B - Source Data 2/Figure 1 - Figure Supplement 1B - Myc.tif]

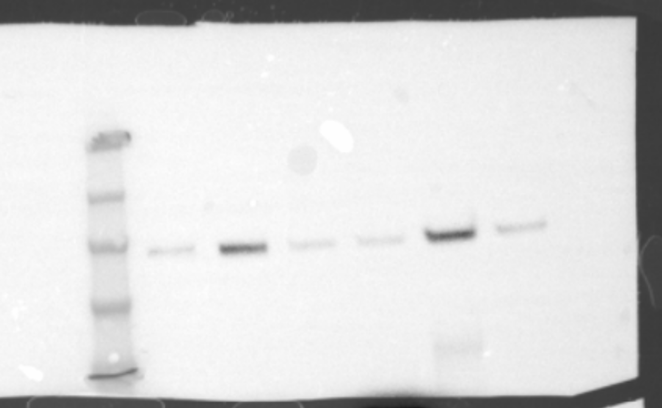

Supplement: Figure 1—figure supplement 1—source data 2. [file elife-106342-fig1-figsupp1-data2.zip › Figure 1 - Figure Supplement 1 Source data 2/Figure 1 - Figure Supplement 1D - Source data 2/Figure 1 - Figure Supplement 1D - Source data 2 - myc.tif]

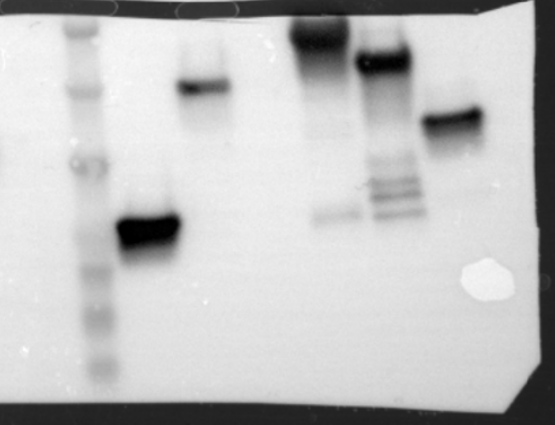

Supplement: Figure 1—figure supplement 1—source data 2. [file elife-106342-fig1-figsupp1-data2.zip › Figure 1 - Figure Supplement 1 Source data 2/Figure 1 - Figure Supplement 1D - Source data 2/Figure 1 - Figure Supplement 1D - Source data 2 - GST.tif]

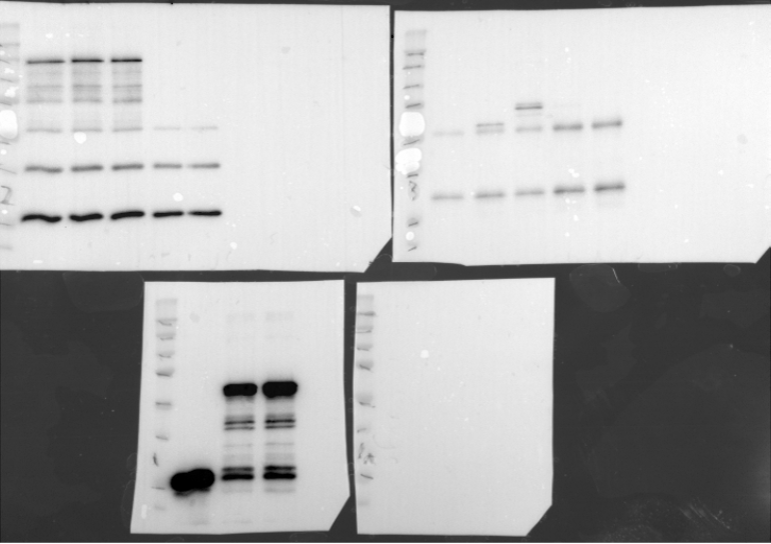

Supplement: Figure 1—figure supplement 1—source data 2. [file elife-106342-fig1-figsupp1-data2.zip › Figure 1 - Figure Supplement 1 Source data 2/Figure 1 - Figure Supplement 1A - Source data 2/Figure 1 - Figure Supplement 1A - Source data 2.tif]

Figure 2 B

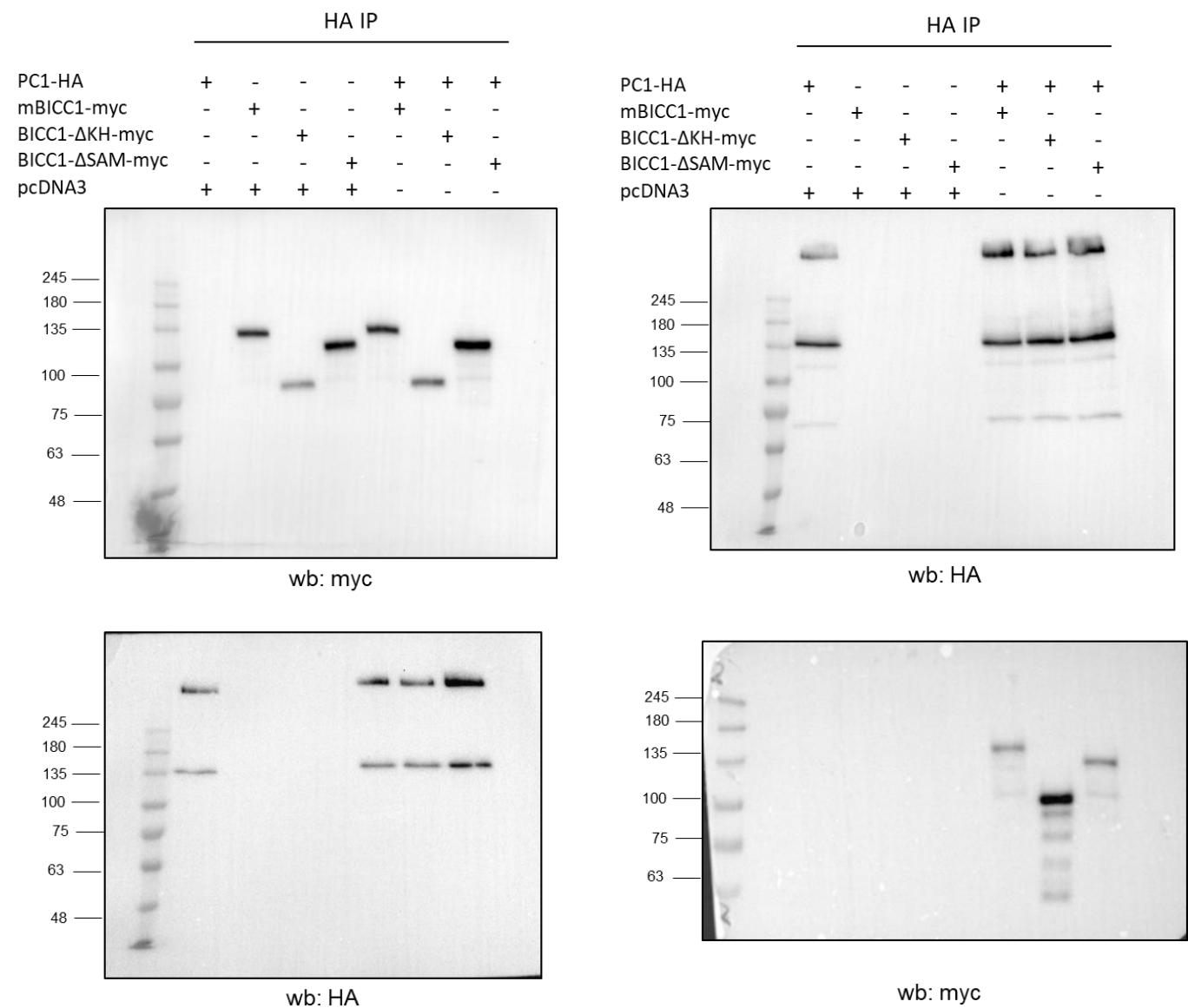

Figure 2, Source Data 2B. Original membranes corresponding to Figure 2, panel B

Supplement: Figure 2—source data 1. [file elife-106342-fig2-data1.zip › Figure 2 Source Data 1/Figure 2 Source Data 2B.pdf]

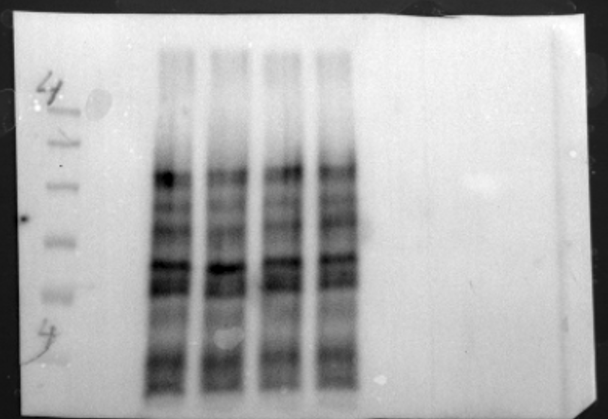

Supplement: Figure 2—source data 2. [file elife-106342-fig2-data2.zip › Figure 2 Source Data 2/Figure 2F Source Data 2/Source data Figure 2F_1.tif]

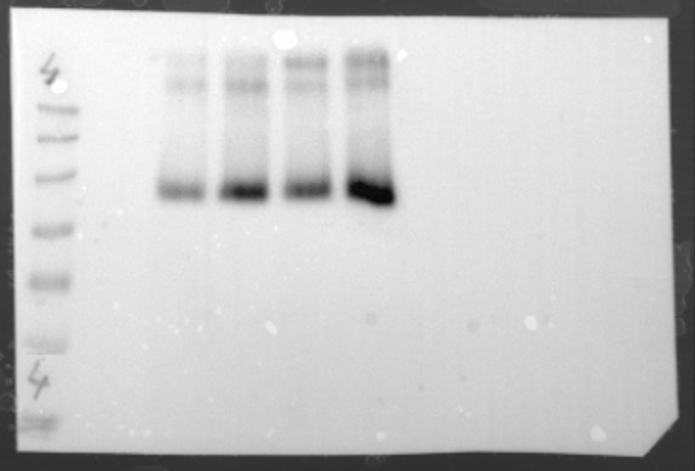

Supplement: Figure 2—source data 2. [file elife-106342-fig2-data2.zip › Figure 2 Source Data 2/Figure 2F Source Data 2/Source data Figure 2F_3.tif]

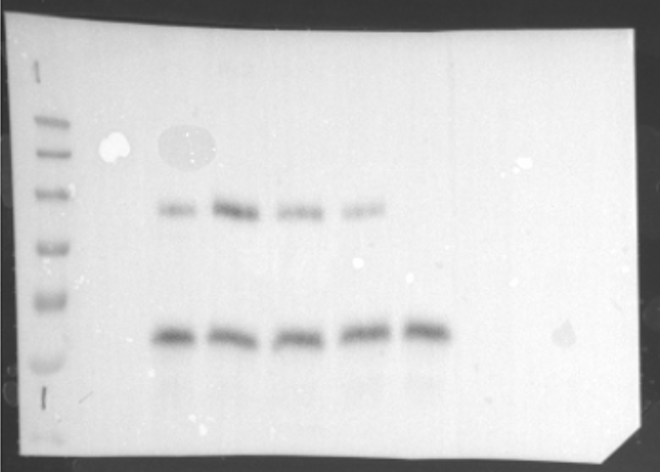

Supplement: Figure 2—source data 2. [file elife-106342-fig2-data2.zip › Figure 2 Source Data 2/Figure 2F Source Data 2/Source data Figure 2F_2.tif]

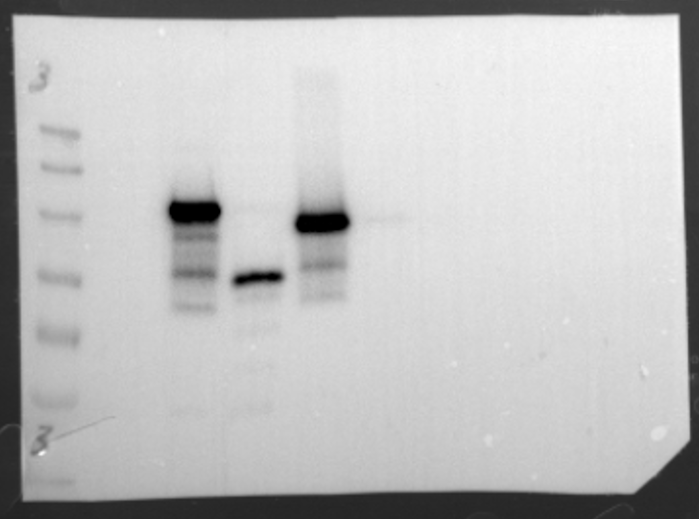

Supplement: Figure 2—source data 2. [file elife-106342-fig2-data2.zip › Figure 2 Source Data 2/Figure 2F Source Data 2/Source data Figure 2F_4.tif]

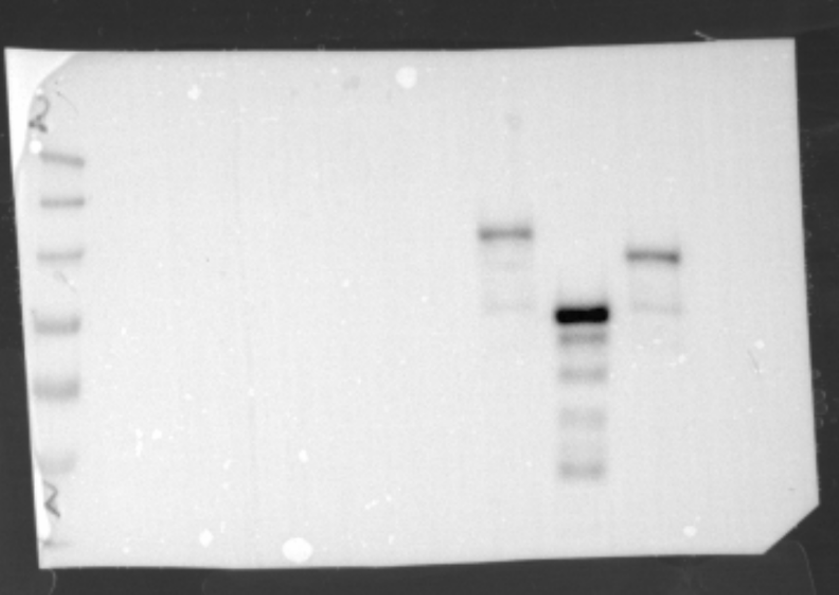

Supplement: Figure 2—source data 2. [file elife-106342-fig2-data2.zip › Figure 2 Source Data 2/Figure 2B Source Data 2/Source data Figure 2B_4.tif]

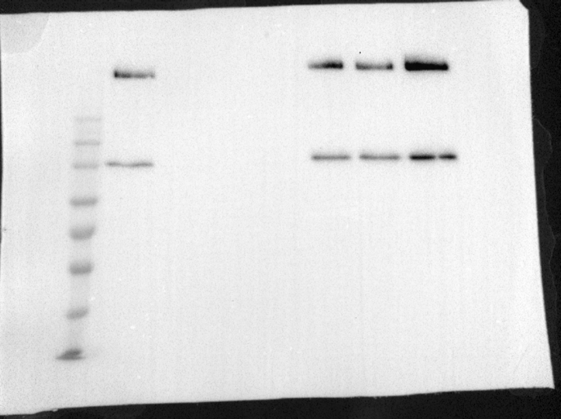

Supplement: Figure 2—source data 2. [file elife-106342-fig2-data2.zip › Figure 2 Source Data 2/Figure 2B Source Data 2/Source data Figure 2B_2.tif]

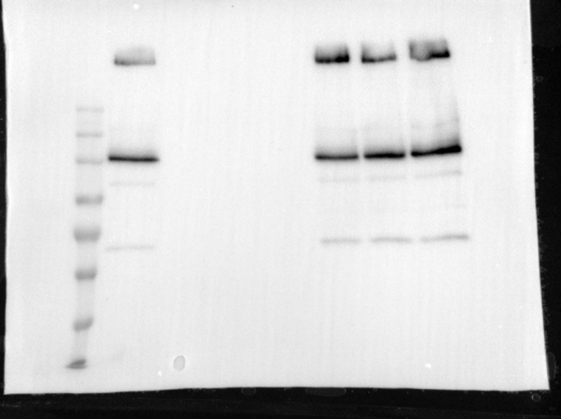

Supplement: Figure 2—source data 2. [file elife-106342-fig2-data2.zip › Figure 2 Source Data 2/Figure 2B Source Data 2/Source data Figure 2B_3.tif]

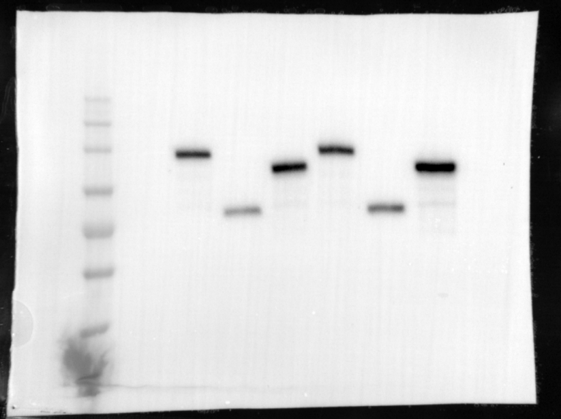

Supplement: Figure 2—source data 2. [file elife-106342-fig2-data2.zip › Figure 2 Source Data 2/Figure 2B Source Data 2/Source data Figure 2B_1.tif]

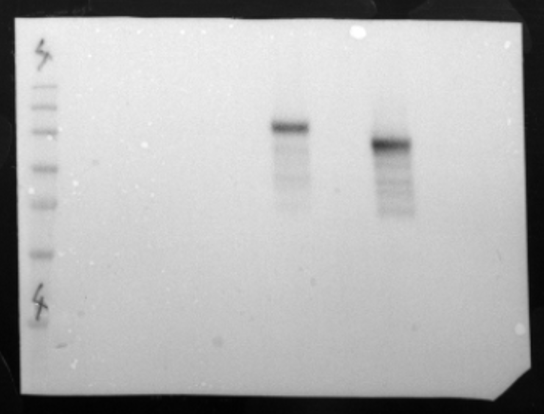

Supplement: Figure 2—source data 2. [file elife-106342-fig2-data2.zip › Figure 2 Source Data 2/Figure 2D Source Data 2/Source data Figure 2D_BICC1 myc pulldown.tif]

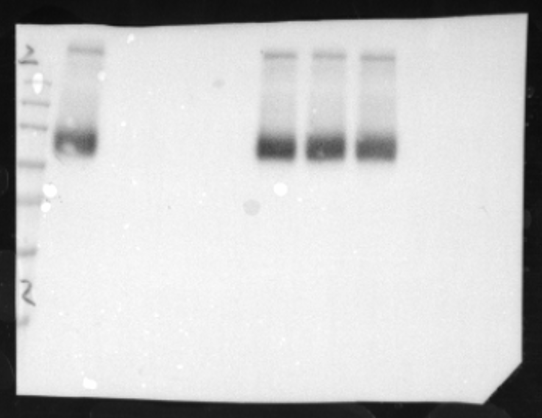

Supplement: Figure 2—source data 2. [file elife-106342-fig2-data2.zip › Figure 2 Source Data 2/Figure 2D Source Data 2/Source data Figure 2D_PC2 lysate.tif]

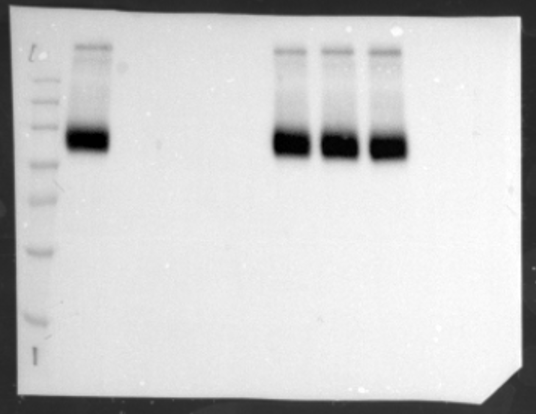

Supplement: Figure 2—source data 2. [file elife-106342-fig2-data2.zip › Figure 2 Source Data 2/Figure 2D Source Data 2/Source data Figure 2D_PC2 pulldown.tif]

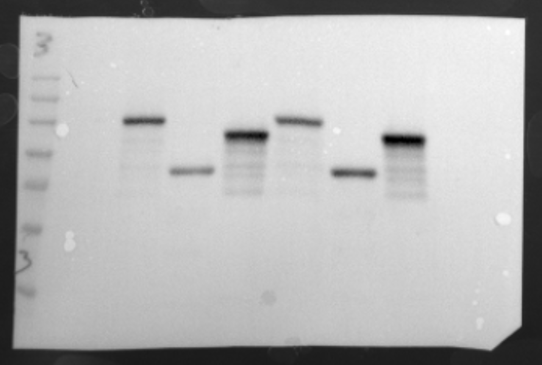

Supplement: Figure 2—source data 2. [file elife-106342-fig2-data2.zip › Figure 2 Source Data 2/Figure 2D Source Data 2/Source data Figure 2D_BICC1 myc lysate.tif]

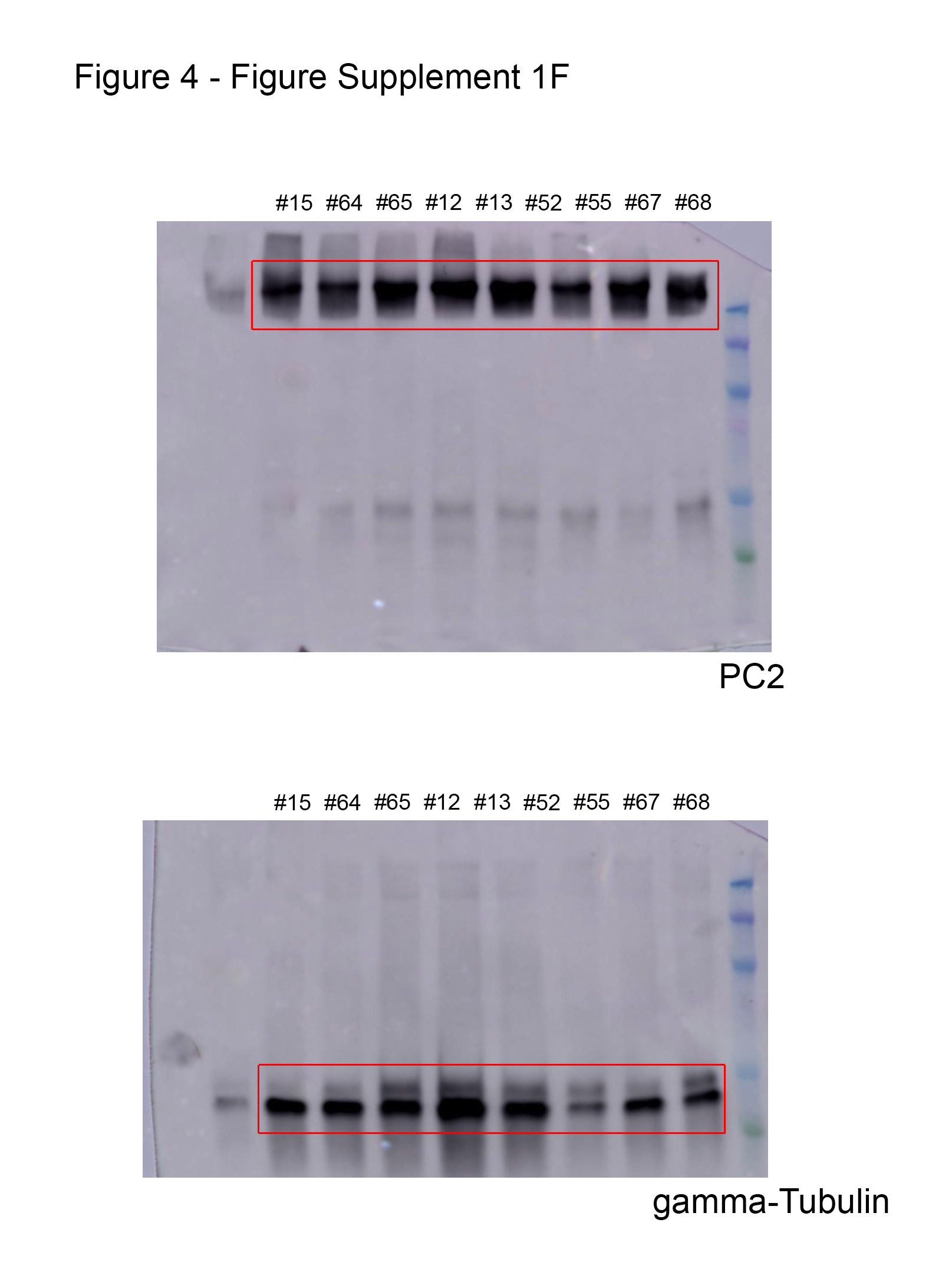

Supplement: Figure 4—figure supplement 1—source data 1. [file elife-106342-fig4-figsupp1-data1.zip › Figure 4 - Figure Supplement 1 Source data 1/Figure 4 - Figure Supplement 1F.tif]

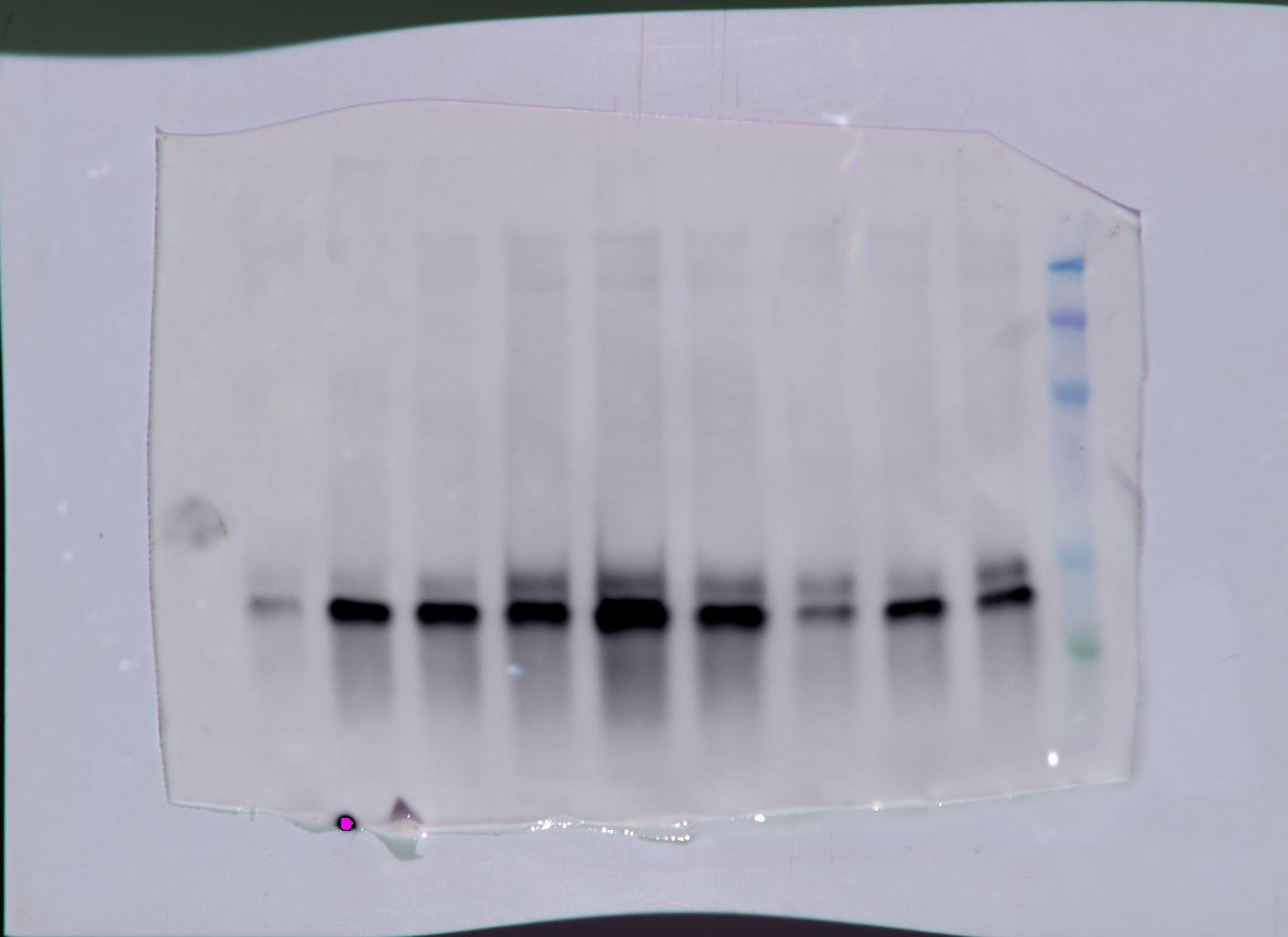

Supplement: Figure 4—figure supplement 1—source data 2. [file elife-106342-fig4-figsupp1-data2.zip › Figure 4 - Figure Supplement 1 Source data 2/Figure 4 - Figure Supplement 1D - Source data 2/g-Tubulin.tiff]

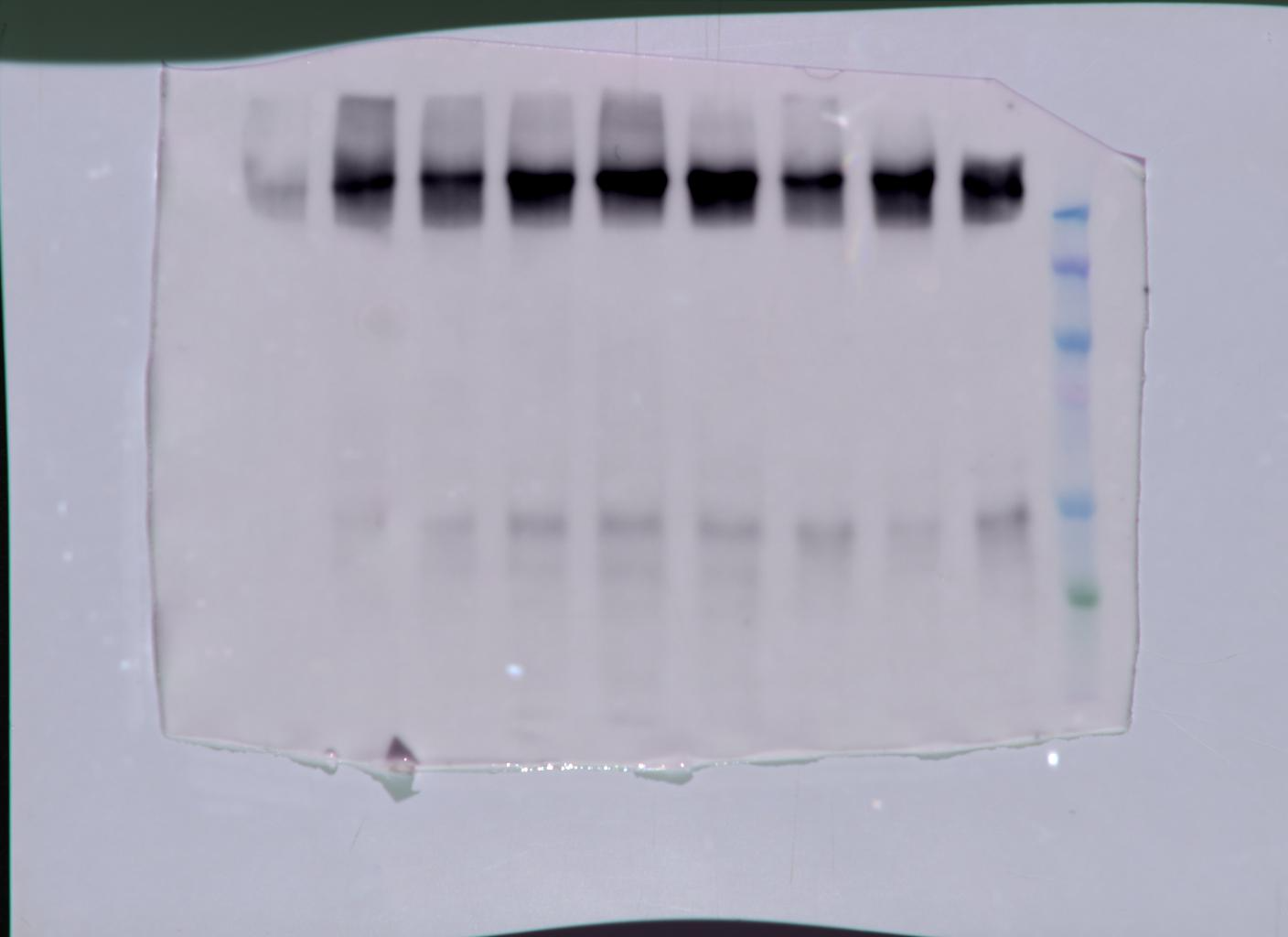

Supplement: Figure 4—figure supplement 1—source data 2. [file elife-106342-fig4-figsupp1-data2.zip › Figure 4 - Figure Supplement 1 Source data 2/Figure 4 - Figure Supplement 1D - Source data 2/PC2.tiff]

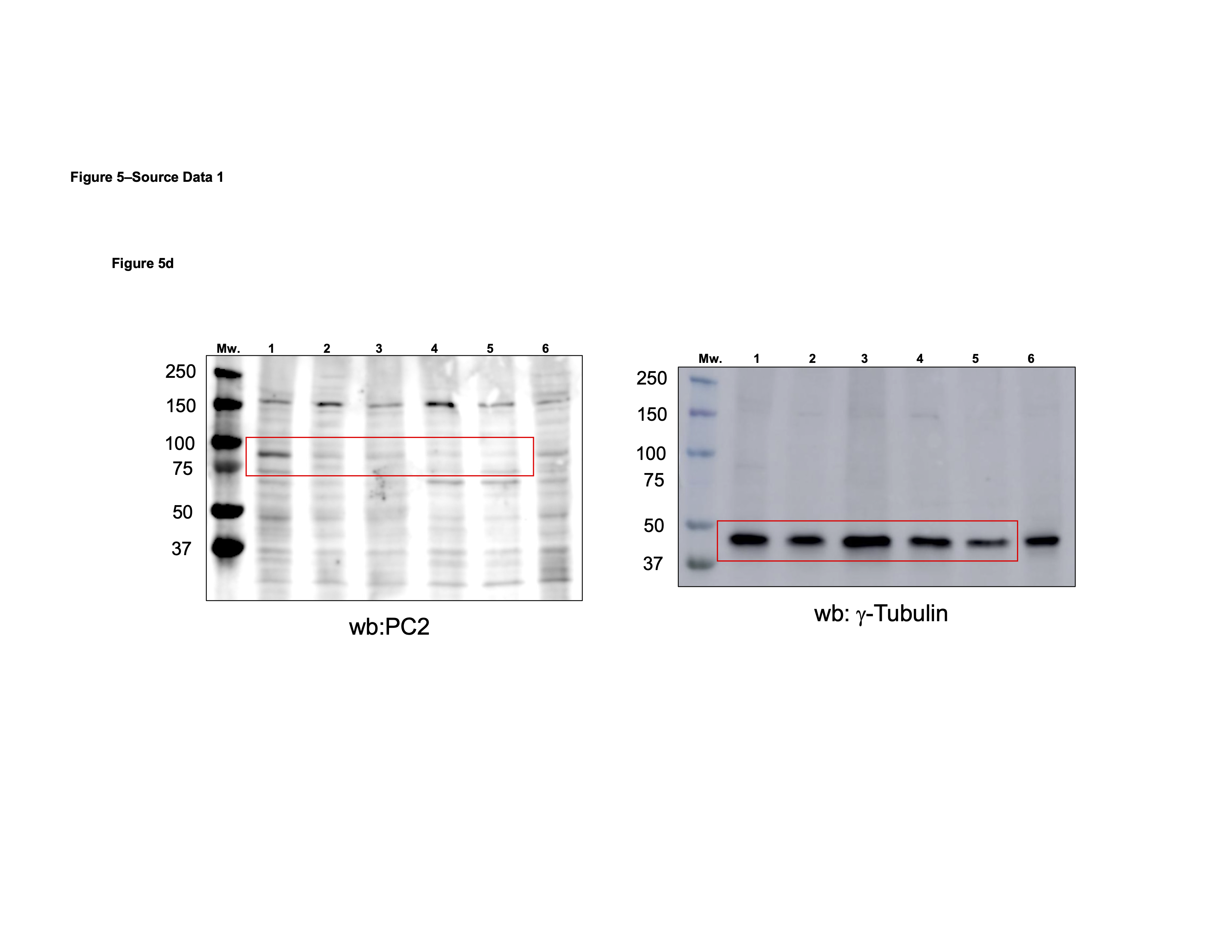

Supplement: Figure 5—source data 1. [file elife-106342-fig5-data1.zip › Figure 5 Source Data 1/Figure 5D Source Data 1.tiff]

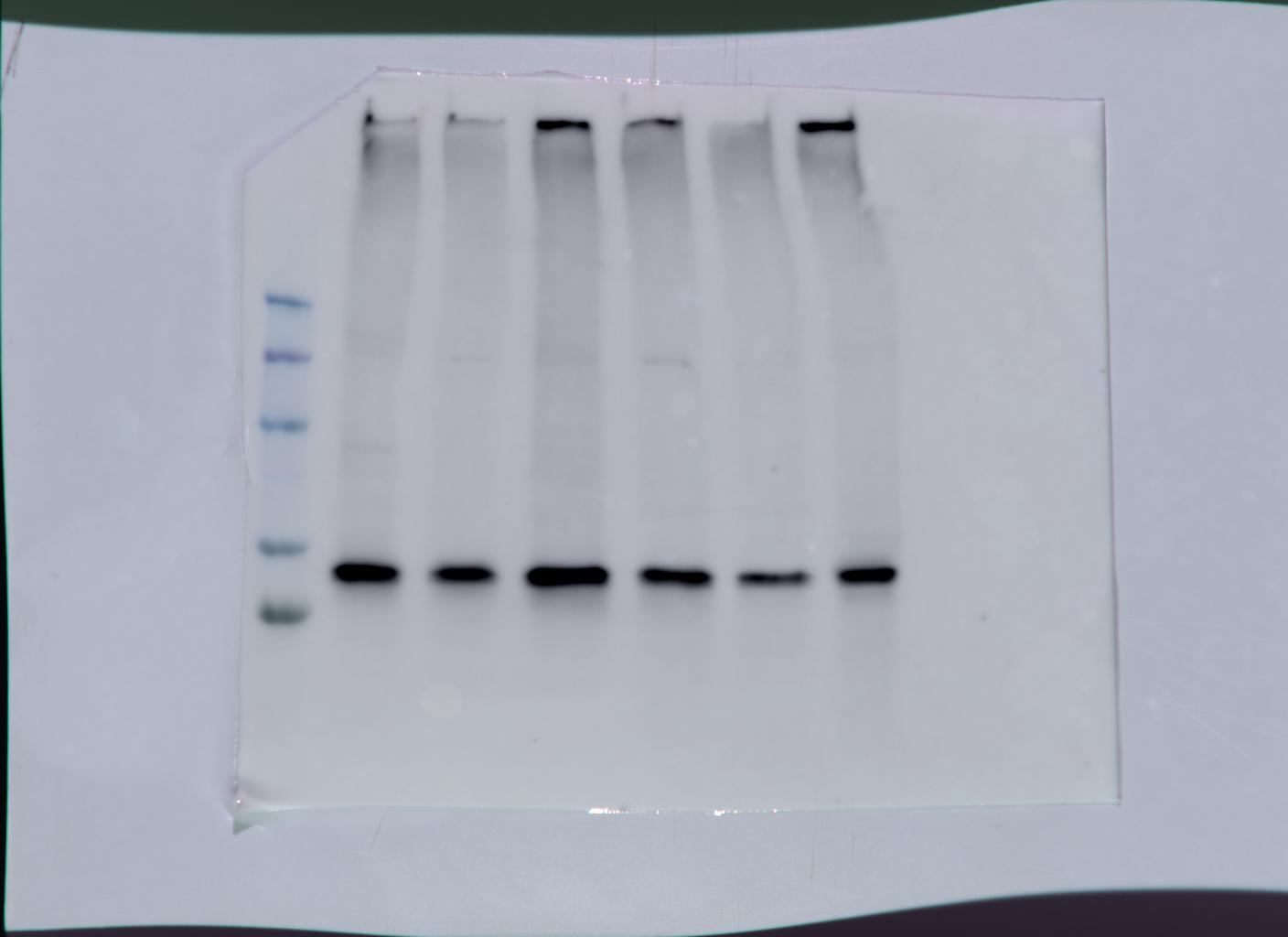

Supplement: Figure 5—source data 2. [file elife-106342-fig5-data2.zip › Figure 5 Source Data 2/Figure 5d Source Data 2/gTubulin.jpg]

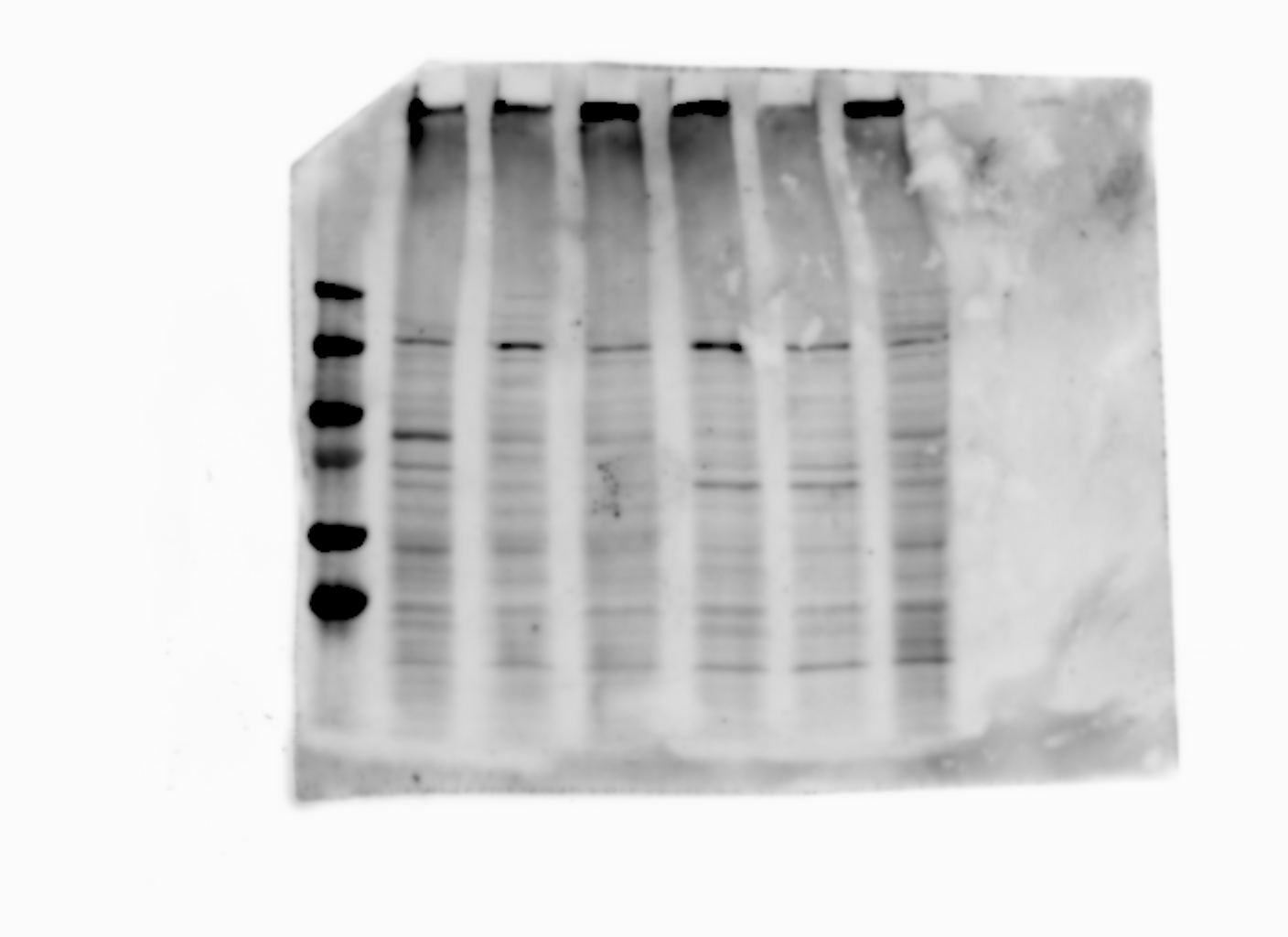

Supplement: Figure 5—source data 2. [file elife-106342-fig5-data2.zip › Figure 5 Source Data 2/Figure 5d Source Data 2/PC2.tif]

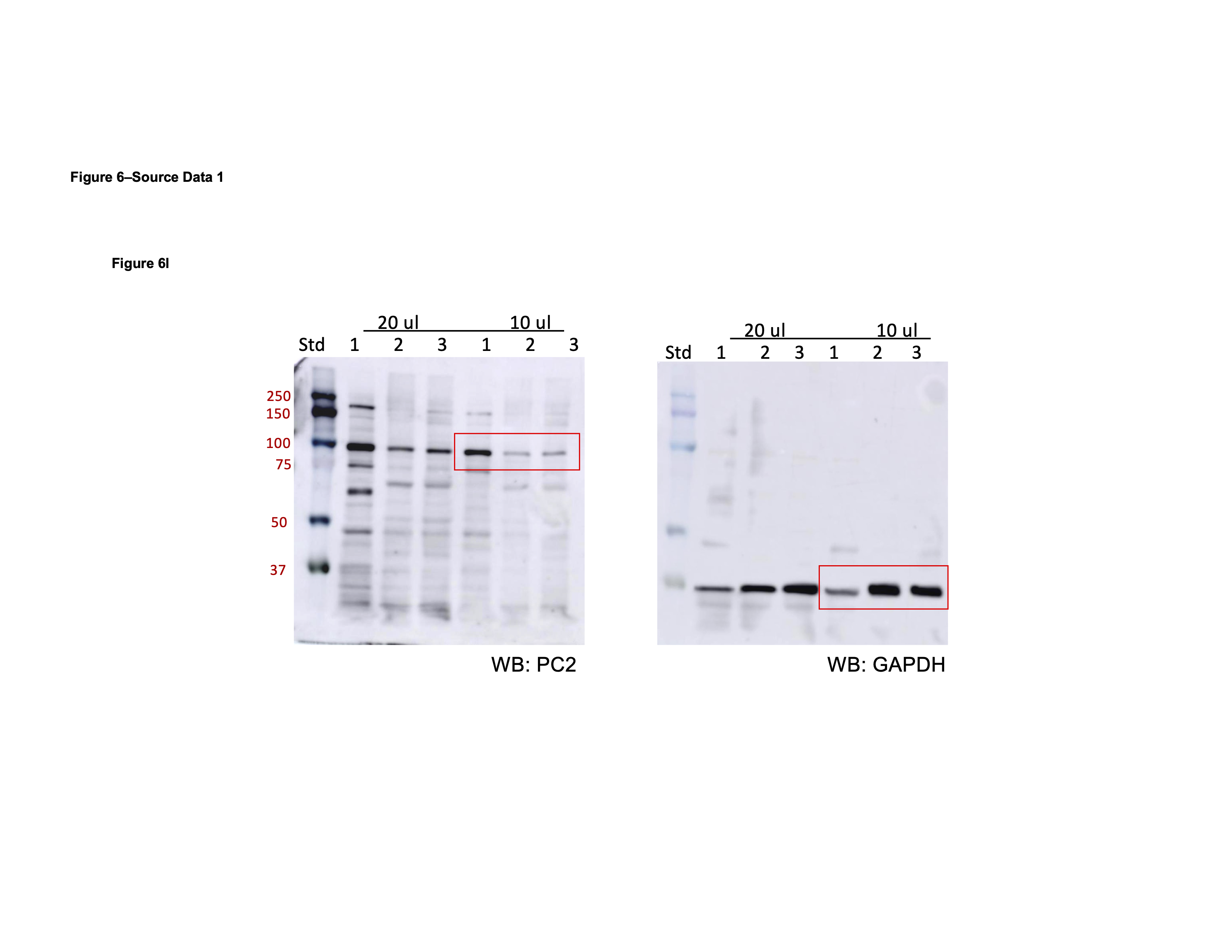

Supplement: Figure 6—source data 1. [file elife-106342-fig6-data1.zip › Figure 6 Source Data 1/Figure 6L Source Data 1.tiff]

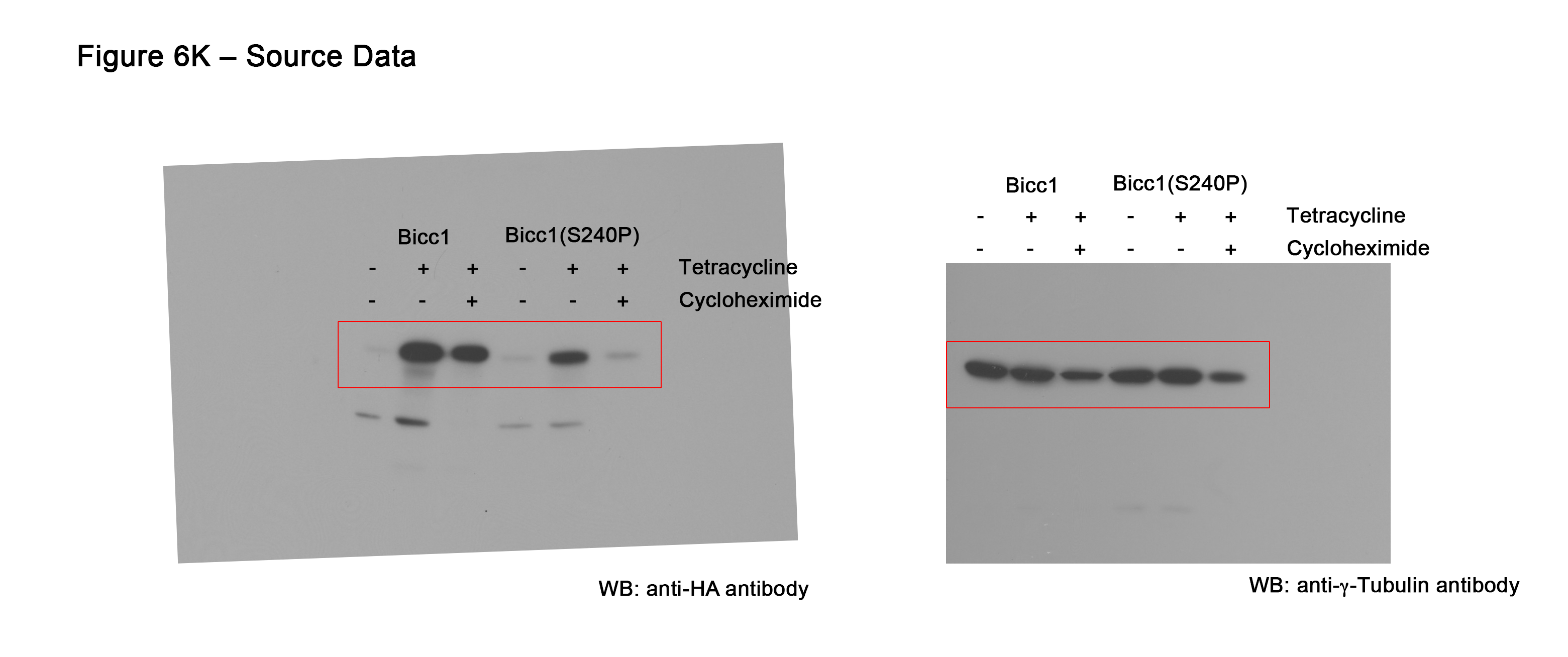

Supplement: Figure 6—source data 1. [file elife-106342-fig6-data1.zip › Figure 6 Source Data 1/Figure 6K Source Data 1.tiff]

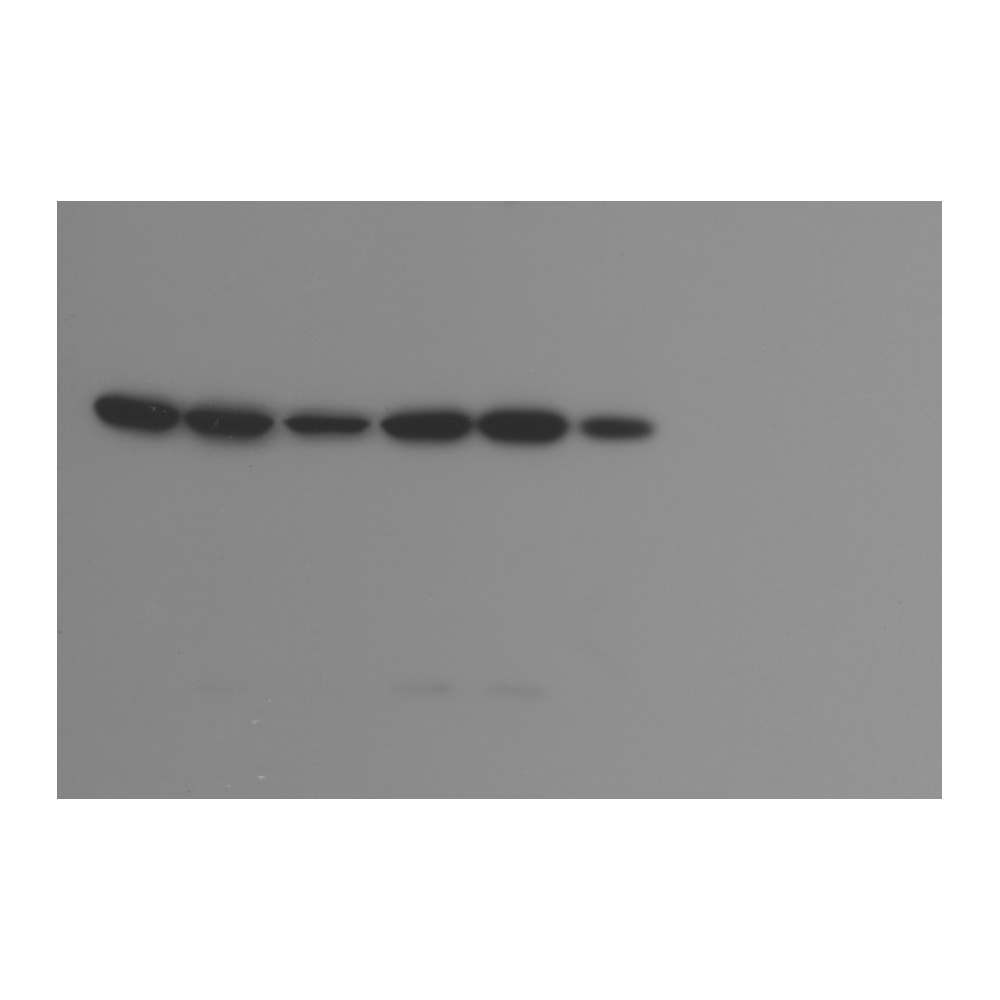

Supplement: Figure 6—source data 2. [file elife-106342-fig6-data2.zip › Figure 6 Source Data 2/Figure 6K Source Data 2/Tubulin.tif]

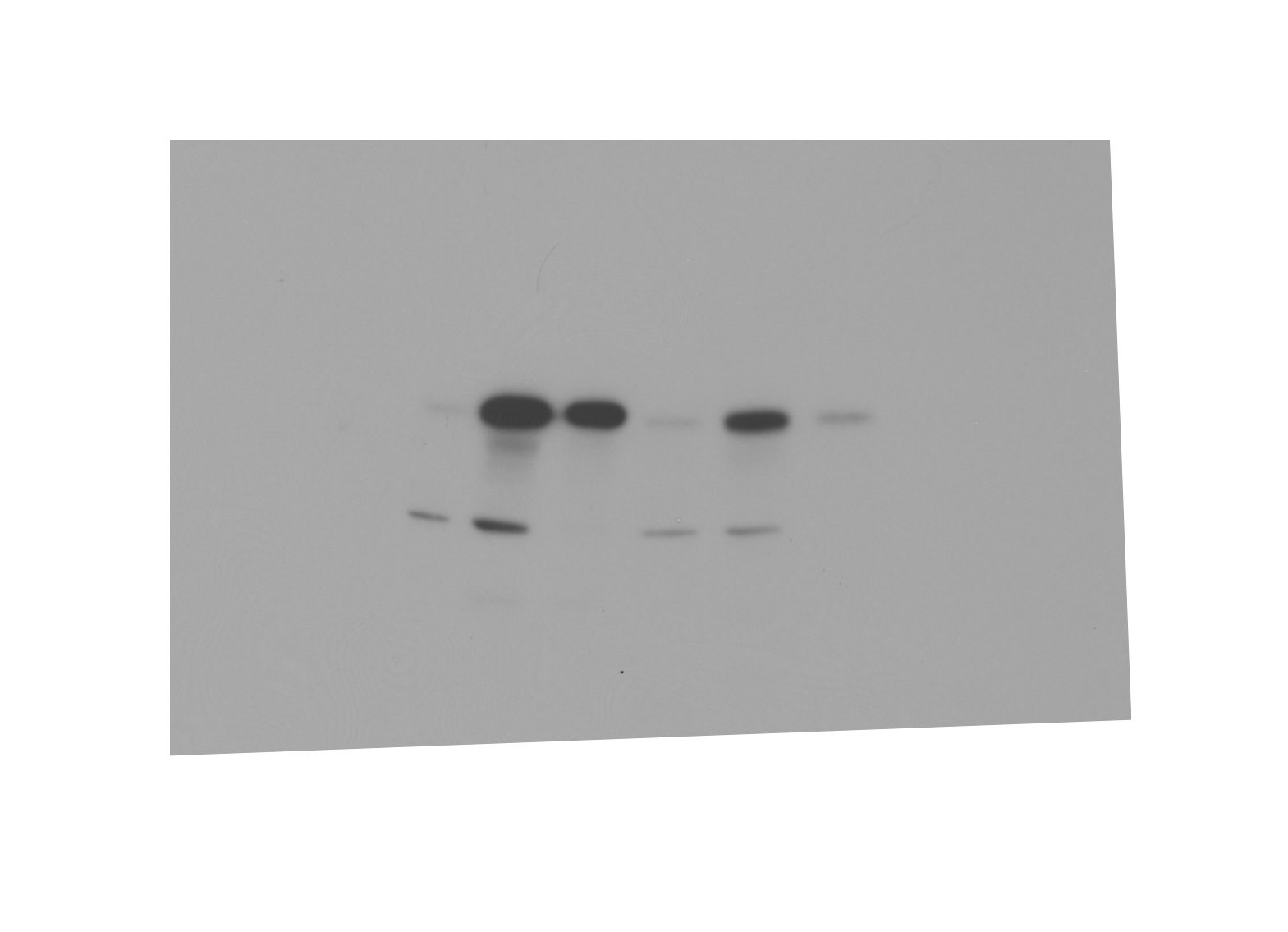

Supplement: Figure 6—source data 2. [file elife-106342-fig6-data2.zip › Figure 6 Source Data 2/Figure 6K Source Data 2/mBicc1.tif]

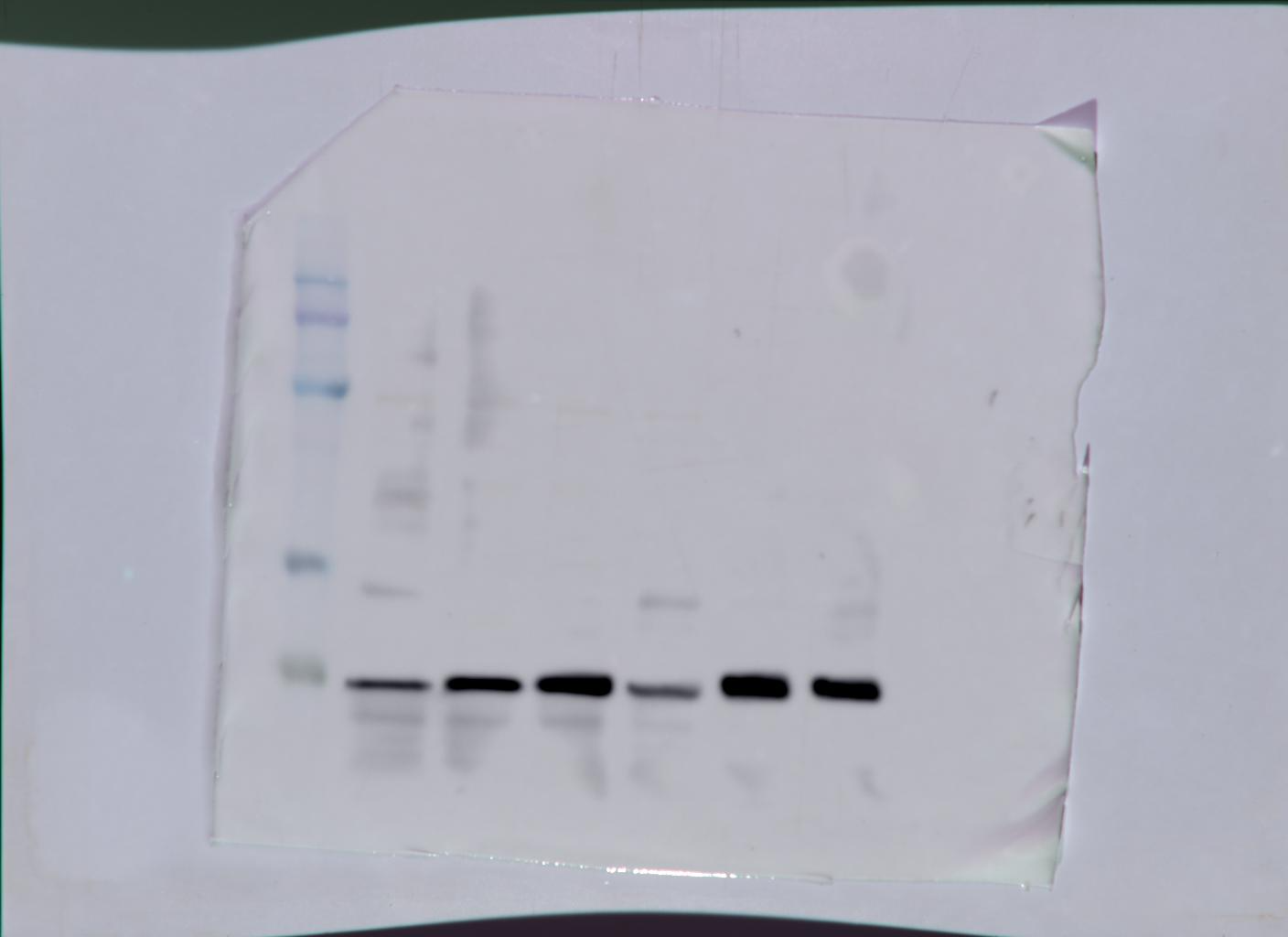

Supplement: Figure 6—source data 2. [file elife-106342-fig6-data2.zip › Figure 6 Source Data 2/Figure 6L Source Data 2/GAPDH.tiff]

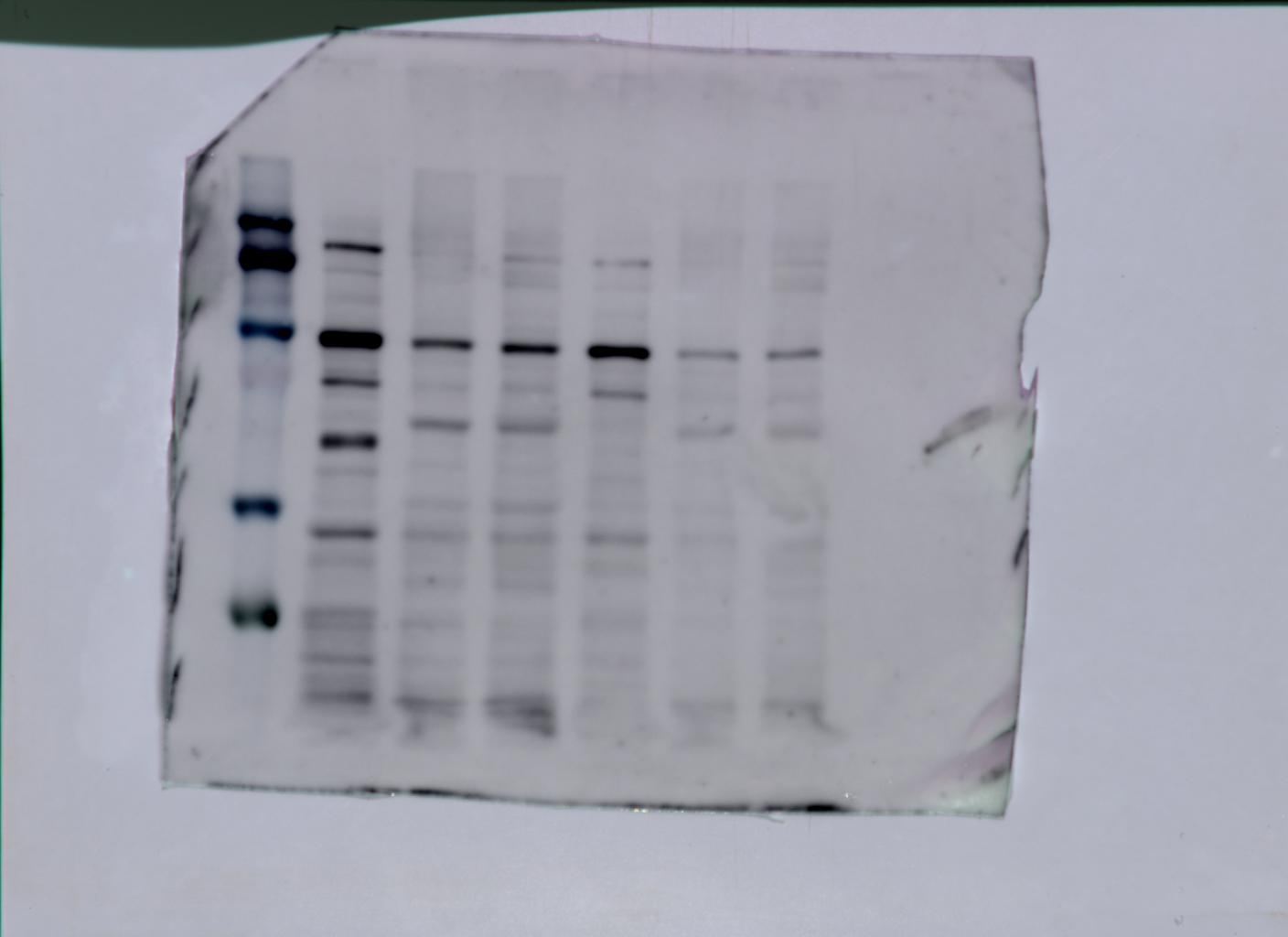

Supplement: Figure 6—source data 2. [file elife-106342-fig6-data2.zip › Figure 6 Source Data 2/Figure 6L Source Data 2/PC2.tiff]
