# Supplementary material for: BICC1 interacts with PKD1 and PKD2 to drive cystogenesis in ADPKD
Source: eLife. 2026 Feb 12;14:RP106342. doi: 10.7554/eLife.106342 (PMC12900513; doi:10.7554/eLife.106342)
Supplement: Figure 2—source data 1. [file elife-106342-fig2-data1.zip › Figure 2 Source Data 1/Figure 2 Source Data 2F.pdf]

Figure 2–Source Data 2F

Figure 2 F

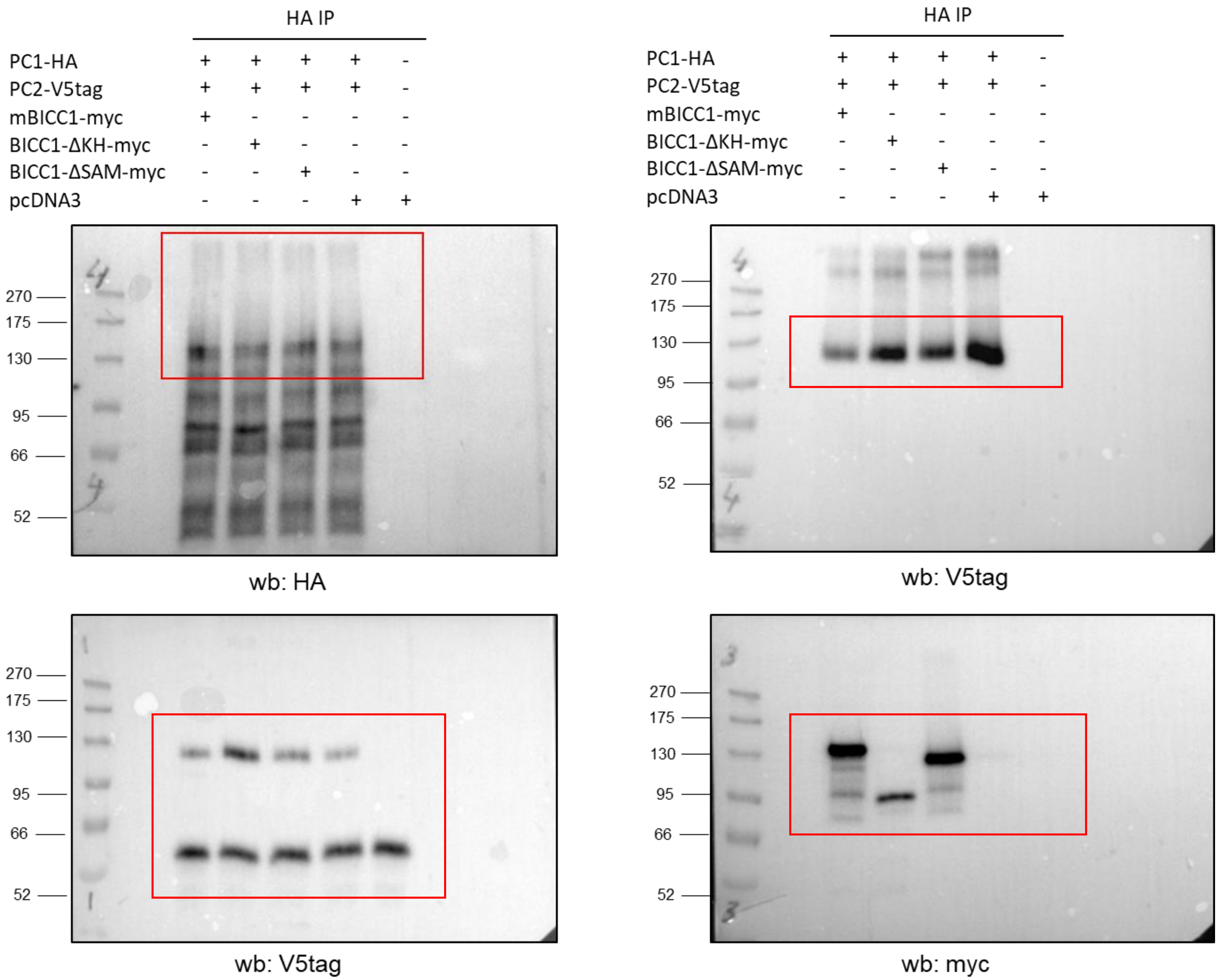

Figure 2, Source Data 2F. Original membranes corresponding to Figure 2, panel F
