# Supplementary material for: BICC1 interacts with PKD1 and PKD2 to drive cystogenesis in ADPKD
Source: eLife. 2026 Feb 12;14:RP106342. doi: 10.7554/eLife.106342 (PMC12900513; doi:10.7554/eLife.106342)
Supplement: Supplementary file 1. — (a) Table of the expected vs. observed frequencies in the Bicc1+/Bpk:Pkd2+/+ x Bicc1+/Bpk:Pkd2+/-crosses at P21. (b) Table of the expected vs. observed frequencies in the Bicc1+/Bpk:Pkd1+/+:Pkhd1-Cre+ x Bicc1+/Bpk:Pkd1+/fl crosses at P14. (c) Table of the in silico analysis of the PKD1 and PKD2 variants identified in VEO-ADPKD patients. (d) Table of the in silico analysis of the BICC1 p.Ser240Pro (S240P) variant. (e) Table of the gene sets enriched in BICC1-KO vs. BICC1-S240P HEK293T cells. [file elife-106342-supp1.docx]

**Supplementary File 1a:** Table of the Expected *vs.* Observed Frequencies in the *Bicc1^+/Bpk^:Pkd2^+/+^* x *Bicc1^+/Bpk^:Pkd2^+/-^* Crosses at P21.

|  | *Bicc1^+/+^:Pkd2^+/+^* | *Bicc1^+/+^:Pkd2^+/-^* | *Bicc1^+/Bpk^:Pkd2^+/+^* | *Bicc1^+/Bpk^:Pkd2^+/-^* | *Bicc1^Bpk/Bpk^:Pkd2^+/+^* | *Bicc1^Bpk/Bpk^:Pkd2^+/-^* |
| --- | --- | --- | --- | --- | --- | --- |
| Observed [N] | 15 | 8 | 29 | 14 | 12 | 9 |
| Observed [%] | 17.2 | 9.2 | 33.3 | 16.1 | 13.8 | 10.3 |
| Expected [%] | 12.5 | 12.5 | 25.0 | 25.0 | 12.5 | 12.5 |

The observed frequency distribution follows the expected Mendelian distribution; A non-Mendelian distribution is not supported by the Pearson’s χ2 test using the Mendel Excel workbook^1^ (p=0.159 using a confidence interval of 0.05). Light gray column is the *Bicc1^Bpk/Bpk^* phenotype, and the dark grey column is the *Bicc1^Bpk/Bpk^:Pkd2^+/-^* phenotype. Note that the compound mutants are at a lower than the expected 12.5% frequency.

**Supplementary File 1b:** Table of the Expected vs. Observed Frequencies in the *Bicc1^+/Bpk^:Pkd1^+/+^: Pkhd1-Cre+* x *Bicc1^+/Bpk^:Pkd1^+/fl^* crosses at P14

|  | *Bicc1^+/+^; Pkd1^+/+^; Pkhd1-Cre−* | *Bicc1^+/+^; Pkd1^+/+^; Pkhd1-Cre+* | *Bicc1^+/Bpk^; Pkd1^+/+^; Pkhd1-Cre−* | *Bicc1^+/Bpk^; Pkd1^+/+^; Pkhd1-Cre+* | *Bicc1^Bpk/Bpk^; Pkd1^+/+^; Pkhd1-Cre−* | *Bicc1^Bpk/Bpk^; Pkd1^+/+^; Pkhd1-Cre+* | *Bicc1^+/+^; Pkd1^+/fl^; Pkhd1-Cre−* | *Bicc1^+/+^; Pkd1^+/fl^; Pkhd1-Cre+* | *Bicc1^+/Bpk^; Pkd1+/fl; Pkhd1-Cre−* | *Bicc1^+/Bpk^; Pkd1+/fl; Pkhd1-Cre+* | *Bicc^Bpk/Bpk;^ Pkd1^+/fl^; Pkhd1-Cre−* | *Bicc1^Bpk/Bpk^; Pkd1^+/fl^; Pkhd1-Cre+* |
| --- | --- | --- | --- | --- | --- | --- | --- | --- | --- | --- | --- | --- |
| Observed (N) | 8 | 2 | 14 | 17 | 4 | 8 | 11 | 18 | 17 | 23 | 6 | 12 |
| Observed (%) | 5.71 | 1.43 | 10 | 12.14 | 2.86 | 5.71 | 7.86 | 12.86 | 12.14 | 16.43 | 4.29 | 8.57 |
| Expected (%) | 6.25 | 6.25 | 12.5 | 12.5 | 6.25 | 6.25 | 6.25 | 6.25 | 12.5 | 12.5 | 6.25 | 6.25 |

The observed frequency distribution does not follow the expected Mendelian distribution; A non-Mendelian distribution is supported by by the Pearson’s χ2 test using the Mendel Excel workbook^1^ (p=0.019 using a confidence interval of 0.05). Light gray columns are the genotypes resulting in a *Bicc1^Bpk/Bpk^* phenotype and the dark grey column is the *Bicc1^Bpk/Bpk^:Pkd1^+/CD^* phenotype. Note that the mutant phenotype is in line with the expected 6.25% frequency.

**Reference:**

1. Montoliu L. Mendel: a simple excel workbook to compare the observed and expected distributions of genotypes/phenotypes in transgenic and knockout mouse crosses involving up to three unlinked loci by means of a chi2 test. *Transgenic Res*. Jun 2012;21(3):677–681. doi:10.1007/s11248-011-9544-4

**Supplementary File 1c:** Table of the *in-silico Analysis of the PKD1 and PKD2 Variants Identified in VEO-ADPKD Patients.*

| **Gene** | ***PKD2*** | ***PKD1*** | ***PKD2*** |
| --- | --- | --- | --- |
| **Chromosomal position** | 4:88046767 | 16:2090945 | 4:88056263 |
| **HGVSc** | c.1445T>G | c.11942C>T | c.1894T>C |
| **HGVSp** | p.Phe482Cys | p.Ala3981Val | p.Cys632Arg |
| **Protein Region** | TRANSMEM-Helical; Range:469-489 | TOPO_DOM-Extracellular; Range:3957-3984 | INTRAMEM-Pore-forming; Range:632-646 |
| **SIFT** | **0.005** | **0.005** | **0.002** |
| **Polyphen2** | 0.519 | **1** | **0.839** |
| **CADD** | **24.9** | **25.9** | **27** |
| **FATHMM** | -0.47 | -0.83 | **-4.45** |
| **Eigen-PC** | 0.450 | 0.356 | **0.538** |
| **GERP++ RS** | **5.61** | 3.12 | **5.36** |
| **EVE** | 0.240 | **0.706** | **0.875** |
| **REVEL** | 0.185 | **0.668** | **0.831** |
| **MetaSVM** | -0.359 | -0.033 | **0.980** |
| **MetaLR** | 0.340 | 0.495 | **0.904** |
| **PrimateAI** | 0.429 | **0.897** | 0.714 |
| **Alphamissense** | 0.088 | **0.503** | **0.993** |
| **ESM1b** | **-7.82** | **-7.96** | **-18.21** |
| **ProtVar** | 1.089 | - | **23.0107** |
| **gnomAD exomes AF** | 0.00204 | 0.00001 | - |
| **gnomAD genomes AF** | 0.00185 | 0.00001 | - |

**Supplementary File 1d:** Table of the *in-silico* Analysis of the *BICC1* p.Ser240Pro (S240P) Variant.

| **p/Change (Domain)** | **Consurf** | **Amino acid (REF/ALT)** | | | | **DynaMut**  **ΔΔG** | **Varsite** | |
| --- | --- | --- | --- | --- | --- | --- | --- | --- |
|  |  | **Polarity** | **Charge** | **Chemical** | **HI** |  | **DP** | **Prediction** |
| S240P (KHL1) | 6 | P/NP | N/N | HYDROXYL/ALIPHATIC | -0.8/1.6 | -0.293 | 1.32 | unfavored |

NP - nonpolar; P - polar; N – neutral; HI - Hydropathy index; ConSurf - conservation scores (9 - conserved, 1 - varia­ble),; ΔΔG in kcal/mol (change in folding free energy between wild-type and mutant structures, ΔΔG ≥ 0 as stabilizing and ΔΔG < 0 as destabilizing); DP - Disease propensity value (normalized ratio of the number of disease‐to‐natural variants of a given type).

**Supplementary File 1e. Table of the Gene Sets Enriched in BICC1-KO *vs.* BICC1-S240P HEK293T Cells.**

| **Rank** | **Geneset** | **NOM p-val** |
| --- | --- | --- |
| 1 | HALLMARK_EPITHELIAL_MESENCHYMAL_TRANSITION | 0 |
| 2 | HALLMARK_UV_RESPONSE_DN | 0.345 |
| 3 | HALLMARK_ANGIOGENESIS | 0.704 |
| 4 | HALLMARK_KRAS_SIGNALING_DN | 0.357 |
| 5 | HALLMARK_MITOTIC_SPINDLE | 0.345 |
| 6 | HALLMARK_FATTY_ACID_METABOLISM | 0.718 |
| 7 | HALLMARK_IL6_JAK_STAT3_SIGNALING | 0.704 |
| 8 | HALLMARK_APICAL_SURFACE | 0.704 |
| 9 | HALLMARK_TNFA_SIGNALING_VIA_NFKB | 0.704 |
| 10 | HALLMARK_NOTCH_SIGNALING | 0.704 |
| 11 | HALLMARK_INTERFERON_GAMMA_RESPONSE | 0.704 |
| 12 | HALLMARK_INTERFERON_ALPHA_RESPONSE | 0.704 |
| 13 | HALLMARK_P53_PATHWAY | 0.704 |
| 14 | HALLMARK_COAGULATION | 0.704 |
| 15 | HALLMARK_MYOGENESIS | 0.704 |
| 16 | HALLMARK_IL2_STAT5_SIGNALING | 0.704 |
| 17 | HALLMARK_APICAL_JUNCTION | 0.704 |
| 18 | HALLMARK_HYPOXIA | 0.704 |
| 19 | HALLMARK_KRAS_SIGNALING_UP | 0.704 |
| 20 | HALLMARK_ANDROGEN_RESPONSE | 0.704 |
| 21 | HALLMARK_APOPTOSIS | 0.704 |
| 22 | HALLMARK_INFLAMMATORY_RESPONSE | 0.704 |
| 23 | HALLMARK_ESTROGEN_RESPONSE_EARLY | 0.704 |
